# Amygdala activity for the modulation of goal-directed behavior in emotional contexts

**DOI:** 10.1371/journal.pbio.2005339

**Published:** 2018-06-05

**Authors:** Kazutaka Maeda, Jun Kunimatsu, Okihide Hikosaka

**Affiliations:** Laboratory of Sensorimotor Research, National Eye Institute, National Institutes of Health, Bethesda, Maryland, United States of America; University of Oxford, United Kingdom of Great Britain and Northern Ireland

## Abstract

Choosing valuable objects and rewarding actions is critical for survival. While such choices must be made in a way that suits the animal’s circumstances, the neural mechanisms underlying such context-appropriate behavior are unclear. To address this question, we devised a context-dependent reward-seeking task for macaque monkeys. Each trial started with the appearance of one of many visual scenes containing two or more objects, and the monkey had to choose the good object by saccade to get a reward. These scenes were categorized into two dimensions of emotional context: dangerous versus safe and rich versus poor. We found that many amygdala neurons were more strongly activated by dangerous scenes, by rich scenes, or by both. Furthermore, saccades to target objects occurred more quickly in dangerous than in safe scenes and were also quicker in rich than in poor scenes. Thus, amygdala neuronal activity and saccadic reaction times were negatively correlated in each monkey. These results suggest that amygdala neurons facilitate targeting saccades predictably based on aspects of emotional context, as is necessary for goal-directed and social behavior.

## Author summary

The amygdala is known to control passive fear responses (e.g., freezing), but it is unclear if it also contributes to active behaviors. To reach certain goals, we (humans and animals) often need to go through fearful environments. We hypothesized that the amygdala contributes to such an active behavior and devised a new foraging task for macaque monkeys in which various emotional contexts changed across many environments. This “exciting” task provoked extremely fast learning and high-capacity memory of objects and environments, and thereby caused extremely fast goal-directed behaviors. We found that the goal-directed behavior was affected by the emotional context in two dimensions (dangerous–safe and rich–poor) separately from the object values. Then, many neurons in the amygdala responded to the environments before any object appeared and did so selectively, depending on the emotional context of the environment. The neuronal activity was tightly correlated with the reaction time of goal-directed behavior across the contexts: faster behavior in dangerous or rich context. These results suggest that the amygdala facilitates goal-directed behavior by focusing on emotional contexts. Such a function is also important for emotional–social behavior and its disorder, including averted eye gaze in autism.

## Introduction

Goal-directed behavior is strongly influenced by the predicted outcome of choices on the basis of repeated experience with multiple objects and actions [1]. However, the outcome of choices often changes, depending on the context [2]. Thus, neurons contributing to goal-directed behavior should integrate information about both behavioral targets and context, which actually involve various brain areas [3–5]. It is thus difficult to understand the mechanism of context specificity: how is context information created, and how is it integrated with target information?

To address these questions, we used visual scenes as contexts, which often serve as “environment contexts” in real life [2]. For a visual scene to establish context, the subject needs to learn the predictable events, for instance, that a robber may appear [6,7]. It is known that various scenes are discriminated in particular areas in the visual cortices based on their visual features [8], but scene context (e.g., dangerous, safe, rich, poor) may be detected in different brain areas where the visual features are associated with the predictable events. One procedure to examine this mechanism is to make multiple scenes that represent a particular context, which would suggest that a context is created regardless of sensory features.

Importantly, goal-directed behavior must start sometime after the subject enters into a particular environment. Thus, the context information can be available earlier than target information, which may allow separate processing mechanisms. To utilize this temporal feature, it would be better if the environment appeared suddenly and unpredictably, which should be followed by, not preceded by, the activation of the context mechanism.

To this end, we created a new foraging task using visual scenes derived from satellite imagery as environments, each of which was presented in a large portion of the subject’s (macaque monkey) visual field. Within each scene, smaller fractal objects appeared, which the subject either reacted to or ignored. Initially, all the visual scenes and objects were novel, but after repeated experiences they started representing several groups of scenes and targets. In one block of the experiment, many scenes were presented randomly so that the context mechanism had to be activated differently each time after the scene appeared.

As the first step in studying the mechanisms of context, we recorded neuronal activity in the amygdala of monkeys performing the foraging task. The amygdala is highly sensitive to emotional stimuli or contexts [3,9,10]. This has been shown clearly by experiments using passive procedures (e.g., Pavlovian conditioning tasks), especially with fearful objects acting as conditioned stimuli [11]. On the other hand, recent studies showed that the amygdala also contributes to goal-directed behavior [12,13]. These studies together raise a hypothesis that the amygdala promotes goal-directed behavior in emotional or dangerous contexts, which is critical in real life. Our study supports this hypothesis, as shown below.

## Results

**Fig 1:**
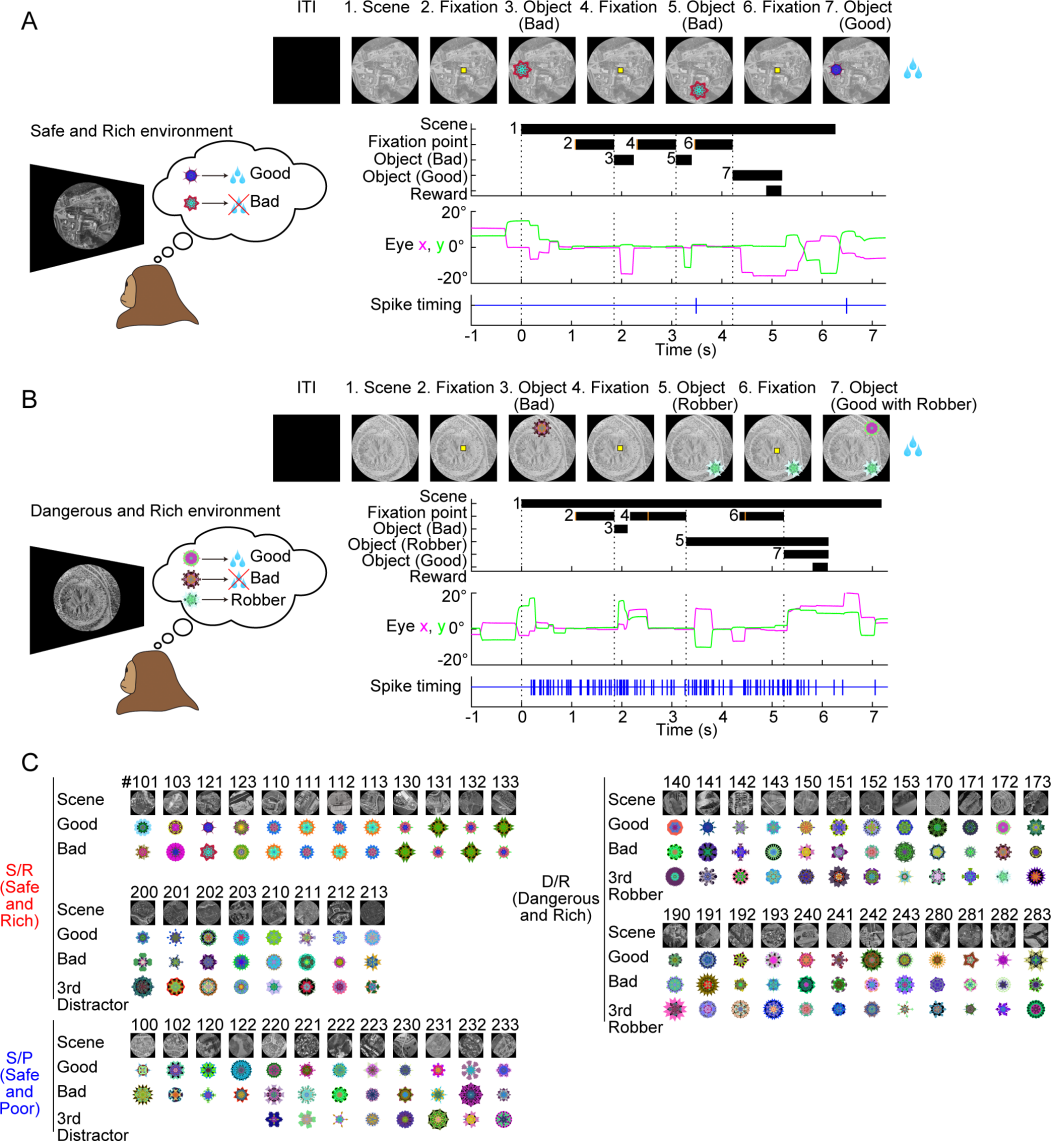
Foraging task in environmental contexts. (**A**) Example trial from monkey PA with a safe environment (#121 in Fig 1C). Top: sequence of events starting with blank screen during the ITI. Bottom: eye position (magenta: horizontal; green: vertical) and spike activity of one amygdala neuron (spike timing, blue). In this trial, the bad object appeared twice, followed by the good object (top). The monkey made saccades from the fixation point to both objects but quickly returned to the center for the bad object and kept fixating the good object to get a reward. The neuron was nearly silent (only two spikes). (**B**) Trial from monkey PA with a dangerous environment (#172 in Fig 1C). After the bad object, the robber object appeared and remained until the good object appeared, to which the monkey made a saccade quickly enough to trigger reward delivery. On other trials when the saccade was delayed, the robber object jumped to the good object and no reward was available. See S1 Fig and Materials and methods for detailed procedures. See also S1 and S2 Movies. The same neuron continued to be active until the reward delivery (unlike in the safe environment in A). Performing these trials correctly required the monkey to retrieve the memories of the objects contained in the scene. We refer to each scene as an “environment.” (**C**) Multiple sets of example scenes (*n* = 56) and objects (*n* = 140) for monkey PA. Each scene contained one good object (associated with a big or small reward) and one bad object (associated with no reward). Some scenes contained a third object that acted as a robber object (D/R) or a distractor object (some in S/R and S/P). The robber object tried to attack the good object and forestall the reward (as in S1 Fig). The distractor remained while another object (good or bad) appeared, but never attacked the other object. Each good object was consistently associated with either a big or a small reward. We thus classified these scenes into three groups. D/R: dangerous (with robber) and rich (big reward). S/R: safe (no robber) and rich (big reward). S/P: safe (no robber) and poor (small reward). The trial continued with objects appearing in random sequence and position until the monkey ended the trial by holding fixation on either the good object (followed by reward) or the bad object (followed by no reward). The scene remained until the end of the trial. These example scene images were derived from OpenAerialMap (https://openaerialmap.org). D/R, dangerous and rich; ITI, intertrial interval; S/P, safe and poor; S/R, safe and rich.

How the monkey performed the foraging task is shown in Fig 1. Initially, the screen in front of the monkey was dark. Next, a large visual scene chosen from satellite imagery appeared suddenly, which acted as an environment. Each scene contained at least two fractal images (“good” and “bad” objects), which appeared one at a time and were randomized both in sequence and position (Fig 1A). In this example trial, the bad object appeared twice, but the monkey avoided it by saccading to it and then quickly looking away (gaze duration <400 ms). Then, the good object appeared, and the monkey chose it by saccading and holding fixation (FX) (gaze duration >400 ms) and obtained a reward. The scene disappeared after the monkey chose either the good object (with reward) or the bad object (with no reward), thus ending the trial. Similar example trials are shown in S1 Movie.

In another trial (Fig 1B), a different scene with different objects appeared. This scene contained a third type of object in addition to good and bad objects, which we call the “robber object.” In this example, the bad object appeared first, which the monkey avoided. Then, the robber appeared and waited for the good object. When the good object appeared, both the monkey and the robber tried to get it first. In this case, the monkey won the competition and got a reward. Had the monkey’s saccade been slower, the robber would have jumped to the good object and stolen the reward (S1 Fig). Similar procedures have been used for rodents [14,15]. These two scenes can be classified as a “safe” scene (i.e., no robber will come; Fig 1A) and a “dangerous” scene (i.e., robber may come; Fig 1B), respectively. Similar example trials are shown in S2 Movie.

Spike activity of one amygdala neuron was recorded during these trials. It was nearly silent (i.e., only fired two spikes) during the safe scene (Fig 1A, bottom) but started firing immediately after the dangerous scene appeared (Fig 1B, bottom). This result raised the possibility that amygdala neurons process scenes selectively.

However, the difference in neuronal activity or behavior could be due to different visual features of the scenes (as described in the Introduction). To address this issue, we created many scenes (together with objects) for each class of environmental context. Fig 1C shows the example scenes used for monkey PA. We classified the scenes into three groups: (1) D/R: dangerous (robber+) and rich (large reward), (2) S/R: safe (robber−) and rich (large reward), and (3) S/P: safe (robber−) and poor (small reward). We did not use the other possible environment, D/P (dangerous and poor) because we found that this combination led the monkeys to quit the task. Based on the three groups, we investigated two dimensions of emotional context, the dangerous–safe dimension (D/R versus S/R) and the rich–poor dimension (S/R versus S/P). These task dimensions roughly correspond to a common way to conceptualizing emotional dimensions, namely valence and arousal [16]. In some of the safe scenes, another type of “distractor object” could appear, which, like the robber, lingered on the screen but never attacked the reward. Task details for trials with distractor and robber objects are shown in S1 Fig.

All three monkeys learned the many combinations of scenes and objects quickly and accurately. S2A Fig shows the change in the correct choice rate (i.e., choosing good objects) when monkey PI learned four new scenes and eight new objects simultaneously. By the end of the first day of learning (13 trials for each scene, 52 total), the correct choice rate approached 100%. After 2–3-d learning sessions, his performance became almost perfect for all the four scenes. Quick learning similarly occurred for the other scenes that monkey PI experienced (*n* = 56), and likewise with monkeys PA and SO (S2B Fig). Average SacRT was initially about 150 ms and quickly decreased to about 100 ms, after the monkey started experiencing the foraging task (S2C Fig). The monkeys’ performance for well-practiced environments remained high after initial learning and during neuronal recording.

**Fig 2:**
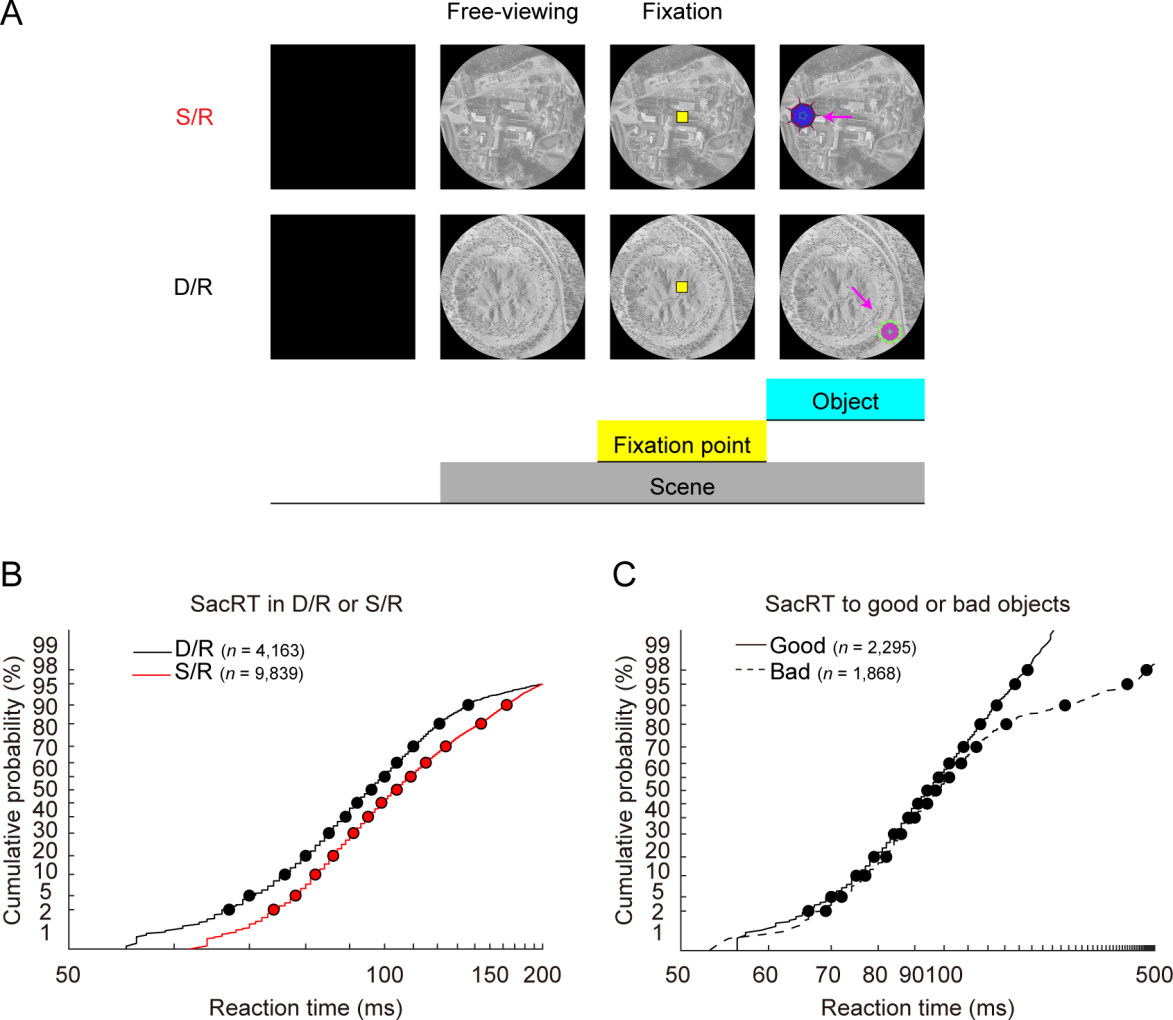
SacRTs in safe and dangerous contexts. (**A**) Safe scene (top, #121 in Fig 1C) and dangerous scene (bottom, #172 in Fig 1C), which appeared occasionally and randomly among many others (Fig 1C). The first object can be a good or bad object, or a robber object (in the dangerous scene). (**B**) Distribution of the SacRT in monkey PA to the first object (good or bad) in the dangerous (D/R) scenes (black) and the safe (S/R) scenes (red). The data are based on all scenes in each group (*n* = 24 for D/R, *n* = 20 for S/R, Fig 1C). SacRT distribution is shown using reciprobit plot, in which a straight line indicates a normal (Gaussian) distribution of the speed of the saccade preparation process [16]. (**C**) SacRT distribution in the dangerous scenes, shown separately for good objects and bad objects. Example scene images were derived from OpenAerialMap (https://openaerialmap.org). D/R, dangerous and rich; S/R, safe and rich; SacRT, saccade reaction time.

However, it was still unclear whether the environment context affected behavior. To address this question, we compared SacRT to good/bad objects between different contexts. Fig 2A shows an example comparison between a safe (S/R) scene (top) and a dangerous (D/R) scene (bottom). We assessed SacRT data in multiple scenes for each context (Fig 1C for monkey PA). We found that SacRT was changed by the environmental contexts (Fig 2B): shorter with dangerous scenes (black) than safe scenes (two-sample *t* test: *P* < 0.001, *t* = −11.086, df = 14,000), even though the reward amount was the same (i.e., big). Notably, the whole distribution of SacRTs (including <100 ms) was shifted between these contexts in monkey PA (Fig 2B). In each context (e.g., dangerous), SacRT was also influenced by the object (Fig 2C): shorter for good objects than bad objects (two-sample *t* test: *P* < 0.001, *t* = −10.385, df = 4,161), but only during the late period (roughly >100 ms). These data suggest that the two factors (scene and object) influenced SacRT and did so with different time courses. It is then likely that there are separate neural mechanisms for the context discrimination and for the object discrimination.

**Fig 3:**
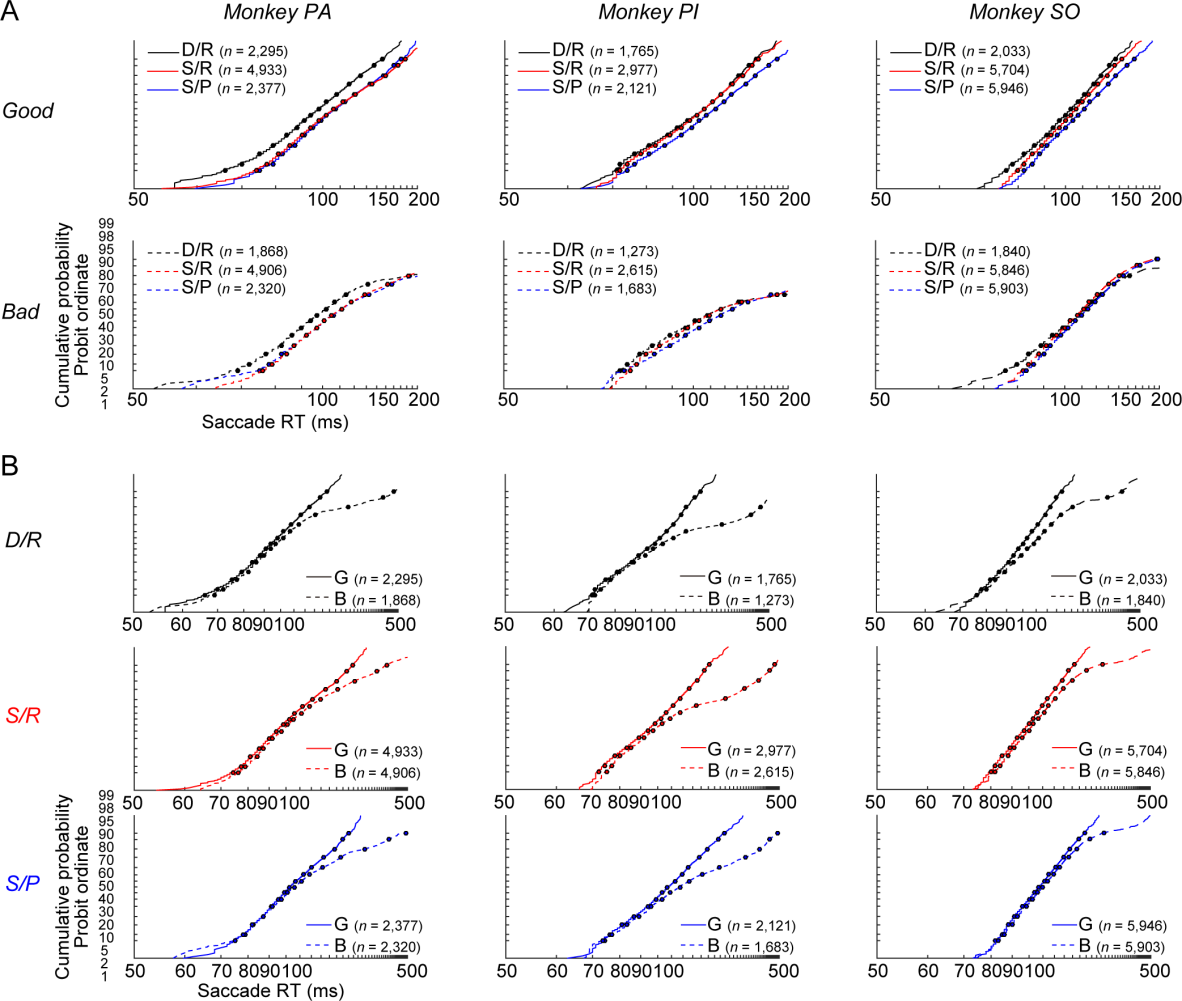
Distribution of SacRTs in different contexts and for different targets. (**A**) Distributions of the SacRT for three groups of scenes are superimposed: D/R, S/R, and S/P (see Fig 1C). They are shown separately for the good and bad objects. Data are shown for monkeys PA (left), PI (center), and SO (right) after learning (>350, >200, and >200 trials for each environment, respectively). (**B**) The same SacRT data are shown separately for the three groups of scenes (D/R, S/R, S/P), but data for good (G) and bad (B) objects are superimposed. The numbers of saccades examined are shown in each graph. The effects of scenes (A) and objects (B) on SacRT were independent and orthogonal in all subjects (two-way ANOVA, monkey PA [scene, F(2,18693) = 68.004, *P* < 0.001; object, F(1,18693) = 381.645, *P* < 0.001; scene*object, F(2,18693) = 5.966, *P* = 0.003], monkey PI [scene, F(2,12428) = 18.313, *P* < 0.001; object, F(1,12428) = 1,216.167, *P* < 0.001; scene*object, F(2,12428) = 21.892, *P* < 0.001], monkey SO [scene, F(2,27266) = 84.198, *P* < 0.001; object, F(1,27266) = 584.585, *P* < 0.001; scene*object, F(2, 27266) = 37.405, *P* < 0.001]). Data used to generate these plots can be found at https://osf.io/2yq8p/?view_only=97c4b290514348bb91cdbb9ec1c85e09. B, bad; D/R, dangerous and rich; G, good; S/P, safe and poor; S/R, safe and rich; SacRT, saccade reaction time.

The SacRT difference between the context and object effects was present in the all monkeys (Fig 3). In order to examine these two factors cleanly, we measured SacRT for the first saccade in a trial (see Fig 2A). Second and subsequent saccades can be influenced by other factors, such as the positions or values of the preceding objects and the presence or absence of the robber/distractor objects. The environmental context affected SacRT across the entire distribution of latencies (Fig 3A), unlike the object value (Fig 3B). In monkey PA (Fig 3A, left), SacRT was shorter with D/R than S/R scenes (two-way ANOVA with scenes and objects, F[2, 18693] = 5.966, *P* = 0.003, post hoc: Tukey–Kramer, *P* < 0.001), indicating that the dangerous–safe dimension had a significant effect on SacRT. In monkey PI (Fig 3A, center), SacRT was shorter with S/R than S/P scenes (F[2, 12428] = 21.892, *P* < 0.001, post hoc: *P* < 0.001), indicating that the rich–poor dimension had a significant effect on SacRT. In monkey SO (Fig 3A, right), SacRT was different in two dimensions of context: (1) dangerous–safe dimension: shorter with D/R than S/R scenes (F[2, 27266] = 37.405, *P* < 0.001; post hoc: *P* < 0.001), and (2) rich–poor dimension: shorter with S/R than S/P scenes (post hoc: *P* < 0.001). These data suggest that SacRT was affected by the context, but somewhat differently in the three subjects. Interestingly, the context effect was observed in saccades to both good and bad objects, even though the monkey left the bad object quickly after making a saccade to it (to avoid no reward). These data again suggest that the context mechanism starts working early after a scene appears, regardless of the upcoming object.

SacRT was also influenced by the object (i.e., shorter for good than bad objects) (Fig 3B) regardless of the scene, but only for the right tail of the distribution (roughly >100 ms). These data suggest two separate neural mechanisms for the scene and object effects. First, the object-processing neurons cannot identify the object’s value immediately after the object appears, until about 100 ms [17]. Second, the scene-processing neurons affect the saccade preparatory process before the object appears, because the scene is already present. That the reciprobit plots showed nearly parallel distributions across environment contexts (Fig 3A) suggests that the speed (rather than threshold) of saccade preparation is changed by the contexts [18], namely faster saccades when the scene was dangerous or rich.

**Fig 4:**
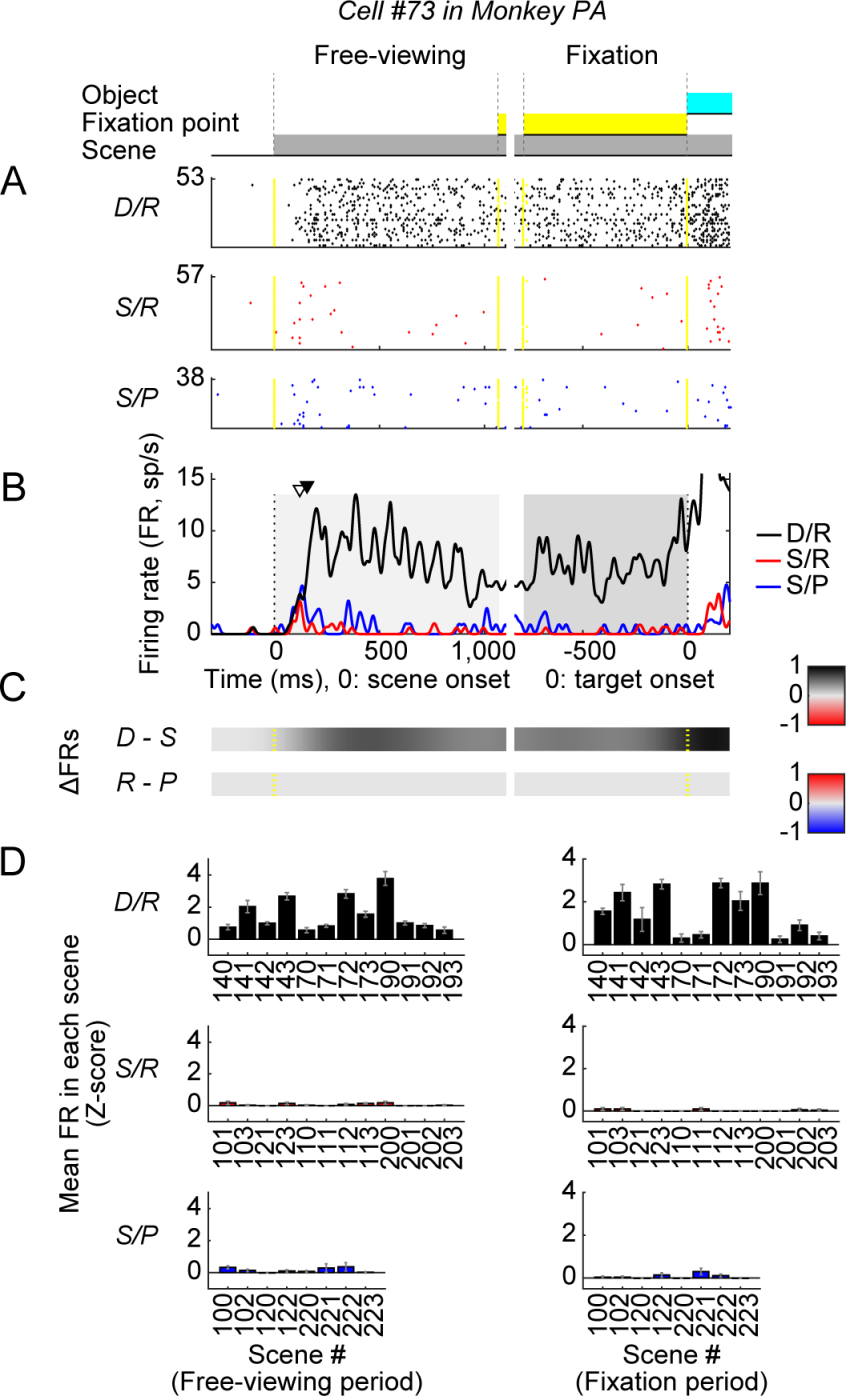
Responses of one example neuron in the amygdala to scene environments. Neuron (#73) in monkey PA that was selectively active in the dangerous context (partially shown in Fig 1). (**A–B**) The neuron’s activities in the three groups of scenes: dangerous (D/R), rich (S/R), poor (S/P). They are shown separately as spike rasters (**A**) and are superimposed as SDF. (**B**). For SDF, each spike was replaced by a Gaussian curve (σ = 10 ms) in this and the following figures. Triangle indicates the onset of the scene response (white) and the onset of the context-dependent differentiation (black). (**C**) Time course of the neuron’s activity bias in two dimensions of context: (1) dangerous–safe (D versus S): difference in activity between D/R and S/R, (2) rich–poor (R versus P): difference in activity between S/R and S/P. In each dimension, the difference of activity (ΔFR score) was calculated in a sliding 300-ms window (10 ms steps) if it was statistically significant (tested by one-way ANOVA and Tukey–Kramer post hoc test); otherwise the score was put as 0. Then, the scores were divided by the maximum of the difference during free-viewing and fixation periods. ΔFR > 0: D > S (top) and R > P (bottom). (**D**) The neuron’s response (z-score) to individual scenes in the three groups examined (see Fig 1C). In this and the following figures, the neuronal data are focused on the activity before the first object appeared (see Fig 1A and 1B). This period was divided into the two parts: free-viewing period (from scene onset to fixation start) (left) and fixation period (from fixation start to object onset) (right). Data used to generate these plots can be found at https://osf.io/2yq8p/?view_only=97c4b290514348bb91cdbb9ec1c85e09. D/R, dangerous and rich; FR, firing rate; S/P, safe and poor; S/R, safe and rich; SDF, spike density function.

These behavioral data suggest that in our foraging task, context-processing neurons should change their activity after the scene appears. Indeed, we found many such neurons in the amygdala. Fig 4 shows the responses of one example neuron in monkey PA to the appearance of many scenes, which were classified in three groups (Fig 4D). This neuron is the same as shown in Fig 1. It started firing in response to D/R scenes but was almost silent when the scene was S/R or S/P. These data suggest that the neuron was sensitive to one dimension of context: dangerous–safe dimension (D/R versus S/R in free-viewing [FV] period, one-way ANOVA, F[2, 29] = 17.932, *P* < 0.001, post hoc: Tukey–Kramer, *P* < 0.001; D/R versus S/R in FX period, one-way ANOVA, F[2, 29] = 18.628, *P* < 0.001, post hoc: Tukey–Kramer, *P* < 0.001) (Fig 4C, dangerous versus safe). It was not significantly sensitive to the other dimension: rich–poor dimension (S/R versus S/P in FV period, post hoc: Tukey–Kramer, *P* = 0.935; S/R versus S/P in FX period, post hoc: Tukey–Kramer, *P* = 0.983) (Fig 4C, rich versus poor). The dangerous–safe difference started quickly, 157 ms after the scene onset (Fig 4B, black triangle). These results suggest that the neuron processed the environmental context in the dangerous–safe dimension.

Alternatively, the difference in the neuronal activity might be caused by the different visual features between the environments. This is one reason we used many visual scenes to represent the same context (Fig 1C). In fact, the neuron’s activity was stronger in the D/R context than the S/R or S/P context regardless of the scene-based differences (assessed in FV periods, one-way ANOVA, F[2, 29] = 17.932, *P* < 0.001; in FX period, one-way ANOVA, F[2, 29] = 18.628, *P* < 0.001) (Fig 4D). Notably, the neuron’s activity was variable across scenes within the same context, which is evident in D/R context (Fig 4D). Its significance will be examined later (S5 Fig).

This neuron was recorded in monkey PA, whose SacRT was shorter in the dangerous than in the safe context (Figs 2 and 3A, left). According to the reciprobit plot [18], this was caused by the difference in speed of the saccade preparation process. To achieve such a speed increase, the saccade generator (e.g., superior colliculus [SC]) should receive modulatory inputs before the preparation process starts. The neuron in Fig 4 may thus contribute to the faster saccades in the dangerous context. Its activity actually further increased toward the end of the FX period (Fig 4B), after which an object appeared and a saccade occurred.

S3 Fig shows the activity of two amygdala neurons in the other monkeys. The first neuron (S3A Fig) was very active spontaneously. It was first inhibited by virtually all scenes (latency: 94 ms). Its activity then became differential (latency: 416 ms), higher with the rich than the poor scenes (S/R versus S/P in FV period, one-way ANOVA, F[2, 29] = 7.003, *P* = 0.003, post hoc: Tukey–Kramer, *P* = 0.015; S/R versus S/P in FX period, one-way ANOVA, F[2, 29] = 8.994, *P* = 0.001, post hoc: Tukey–Kramer, *P* = 0.001), but not different between the dangerous and safe scenes (D/R versus S/R in FV period, *P* = 0.783; D/R versus S/R in FX period, *P* = 0.747). This neuron was recorded in monkey PI, whose SacRT was shorter with the rich than the poor scenes (Fig 3A, center). The neuron may thus contribute to the faster saccades in the rich context.

The second neuron (S3B Fig) was first activated by virtually all scenes (latency: 76 ms). Its differential activity evolved later (latency: 201 ms for S/P and 1,662 ms for D/R in comparison with S/R) in two dimensions, namely (1) dangerous–safe dimension (D/R versus S/R in FV period, one-way ANOVA, F[2, 29] = 4.407, *P* = 0.021, post hoc: Tukey–Kramer, *P* = 0.993; D/R versus S/R in FX period, one-way ANOVA, F[2, 29] = 17.229, *P* < 0.001, post hoc: Tukey–Kramer, *P* = 0.008) and (2) rich–poor dimension (S/R versus S/P in FV period, post hoc: Tukey–Kramer, *P* = 0.030; S/R versus S/P in FX period, post hoc: Tukey–Kramer, *P* = 0.018). This neuron was recorded in monkey SO, whose SacRT was different in the same two dimensions: (1) D/R < S/R and (2) S/R < S/P. The neuron may thus contribute to the faster saccades in the dangerous and rich contexts.

**Fig 5:**
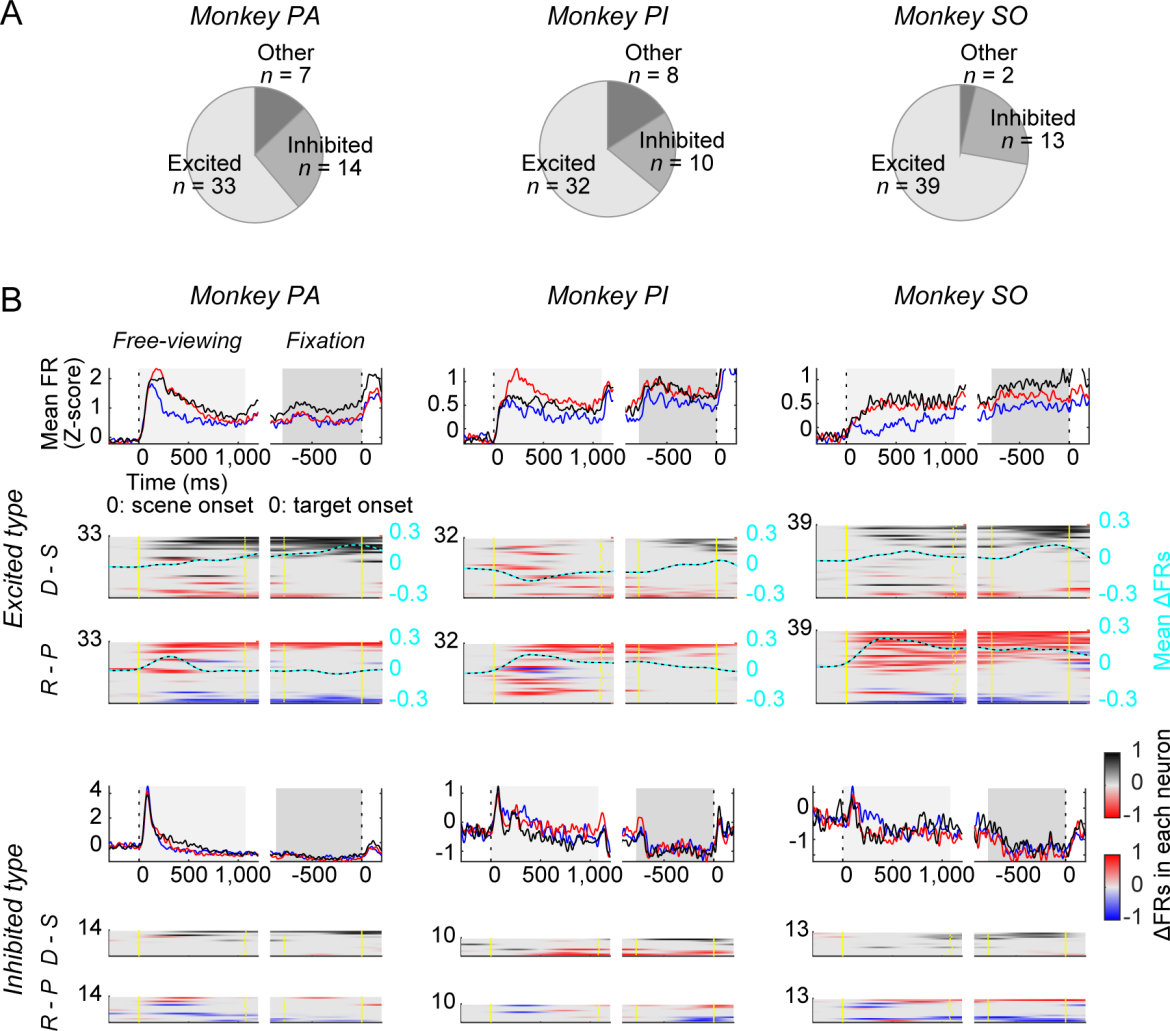
Responses of amygdala neurons in three monkeys to scene environments. (**A**) The numbers of neurons (excited type, inhibited type, others) in monkeys PA, PI, and SO. (**B**) Average activities in the three groups of environments: dangerous (D/R), rich (S/R), and poor (S/P). They are shown separately for the excited-type neurons (top) and the inhibited-type neurons (bottom) for each monkey. The averaging was based on the normalized z-scores of individual neurons (see Materials and methods). Shaded gray area indicates the free-viewing (left) and fixation (right) periods. (**B**, bottom) Time course of the activity biases of individual neurons in two dimensions of environmental context: dangerous–safe dimension (D versus S) and rich–poor dimension (R versus P) (same format as Fig 4C). Individual neuron data are sorted by their mean ΔFR scores in the fixation period, high to low scores. In each dimension, the averaged ΔFR scores are shown by a cyan line with black dots. Data used to generate these plots can be found at https://osf.io/2yq8p/?view_only=97c4b290514348bb91cdbb9ec1c85e09. D/R, dangerous and rich; FR, firing rate; S/P, safe and poor; S/R, safe and rich.

The combined activity of amygdala neurons in monkeys PA, PI, and SO is shown in Fig 5. After the scene appeared, a majority of the neurons increased their activity (excited type, Fig 5B top), while some neurons eventually decreased their activity (inhibited type, Fig 5B bottom) (Fig 5A). Many of them were activated (or inhibited) immediately after the scene appeared (S4 Fig). Their activity then diverged depending on the context: dangerous–safe dimension (D versus S) and/or rich–poor dimension (R versus P). This occurred more clearly among excited-type neurons (Fig 5B, top). The context effect sometimes changed between the early FV period and the late FX period. During the FV period, the neuronal activity tended to be higher in the rich (red) than poor (blue) context, which was statistically significant in all three monkeys (one-way ANOVA and Tukey–Kramer post hoc test; PA: F[2, 53] = 4.984, *P* = 0.01, post hoc, *P* = 0.019; PI: F[2, 53] = 6.937, *P* = 0.002, post hoc, *P* = 0.002; SO: F[2, 29] = 103.568, *P* < 0.001, post hoc, *P* < 0.001). The significant sensitivity to the dangerous–safe dimension (i.e., black versus red) emerged later in the FX period in two monkeys, PA (F[2, 53] = 25.924, *P* < 0.001, post hoc, *P* < 0.001) and SO (F[2, 29] = 51.747, *P* < 0.001, post hoc, *P* < 0.001). Inhibited-type neurons (Fig 5B, bottom) overall were clearly inhibited only during the late period (FX). Their inhibitory responses were not clearly related to the context-dependent modulation of SacRT (PA in FV: F[2, 53] = 6.569, *P* = 0.003 [D versus S: *P* = 0.017; R versus P: *P* = 0.753]; PA in FX: F[2, 53] = 0.868, *P* = 0.426 [D versus S: *P* = 0.449; R versus P: *P* = 0.995]; PI in FV: F[2, 53] = 4.846, *P* = 0.012 [D versus S: *P* = 0.014; R versus P: *P* = 0.933]; PI in FX: F[2, 53] = 2.239, *P* = 0.117 [D versus S: *P* = 0.167; R versus P: *P* = 0.994]; SO in FV: F[2, 29] = 2.916, *P* = 0.070 [D versus S: *P* = 0.558; R versus P: *P* = 0.056]; SO in FX: F[2, 29] = 5.427, *P* = 0.010 [D versus S: *P* = 0.008; R versus P: *P* = 0.143]), unlike excited type neurons.

**Fig 6:**
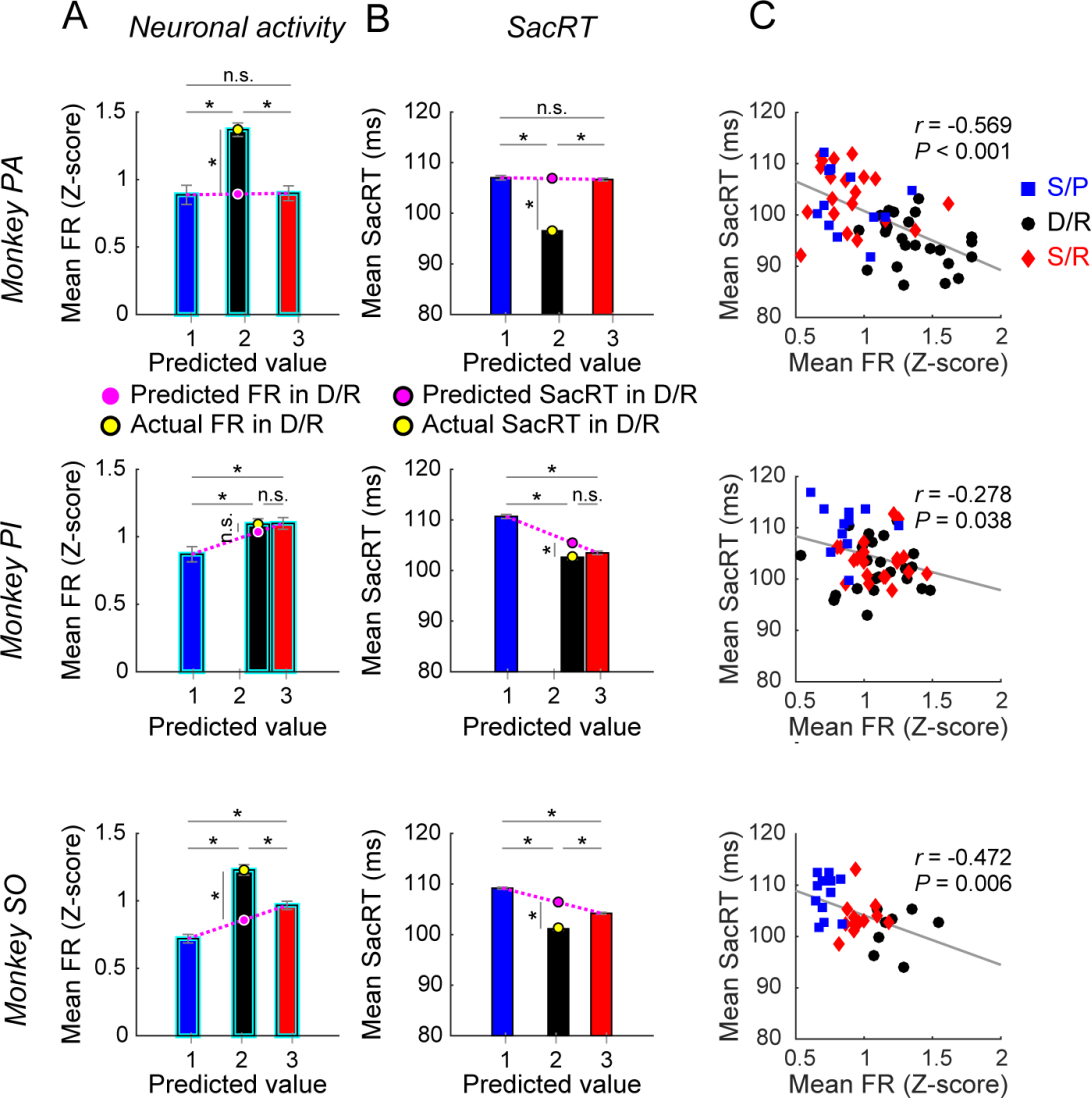
Amygdala neuronal activity and SacRT modulated by two dimensions of scene context. (**A–B**) Neuronal activity during the fixation period (**A**) and SacRT (**B**) in three monkeys (PA, PI, SO), shown separately for three groups of scenes: S/P, D/R, and S/R. The data (ordinate) are plotted against the predicted reward value (abscissa), which was defined as: reward amount × success rate (see Materials and methods). Neuronal activity was based on the normalized z-scores; SE is shown by a vertical bar. Asterisk (*) indicates statistically significant contrasts at *P* < 0.05 (scene context: one-way ANOVA, post hoc: Tukey–Kramer [see S1 Table]; predicted versus actual in D/R: one-sample *t* test). (**C**) Relation between the neuronal activity (abscissa) and SacRT (ordinate) for individual scenes. Statistics (Pearson’s correlation) are shown in each graph. The color of each data point indicates the scene context, as used in A and B. Data are based on excited-type neurons (Fig 5). Data used to generate these plots can be found at https://osf.io/2yq8p/?view_only=97c4b290514348bb91cdbb9ec1c85e09. D/R, dangerous and rich; S/P, safe and poor; S/R, safe and rich; SacRT, saccade reaction time.

These data raise the possibility that amygdala neurons, as a population, affect saccadic eye movements based on the rich–poor and dangerous–safe dimensions of context. To further address this question, we compared the neuronal activity during the FX period and SacRT (Fig 6A and 6B), because the FX period is immediately before the saccade (Fig 2A). The effect of the rich–poor context was evaluated by the difference between the poor (S/P) scenes (blue) and the rich (S/R) scenes (red). In monkeys PI and SO, neuronal activity was higher, while SacRT was shorter with the rich than poor scenes (one-way ANOVA and Tukey–Kramer post hoc test; activity in PI: *P* = 0.006; activity in SO: *P* < 0.001; SacRT in PI: *P* < 0.001; SacRT in SO: *P* < 0.001, S1 Table).

Additionally, the dangerous (D/R) scenes (black) affected the neuronal activity and SacRT. In monkeys PA and SO, neuronal activity with the dangerous (D/R) scenes was higher than the safe scenes, either rich (S/R) or poor (S/P) scenes (activity in PA: *P* < 0.001; activity in SO: *P* < 0.001), while SacRT was shorter than the safe scenes (SacRT in PA: *P* < 0.001; SacRT in SO: *P* < 0.001).

In Fig 6A and 6B, the neuronal activity and SacRT are plotted against the predicted reward value. Although the reward volume per delivery event was the same between the dangerous and rich scenes (S1A Fig), the predicted reward value was smaller with the dangerous scenes because the reward was sometimes removed by the robber object (S1C Fig). Then, the effect of dangerous context (yellow circle) can be estimated by comparing the safe context with the same predicted reward value (pink circle). According to this analysis, even in monkey PI, the neuronal activity tended to be higher (one-sample *t* test, PA: *P* < 0.001, *t* = 9.628; PI: *P* = 0.22, *t* = 1.261; SO: *P* < 0.001, *t* = 6.518) and SacRT shorter (one-sample *t* test, PA: *P* < 0.001, *t* = −31.155; PI: *P* < 0.001, *t* = −6.863; SO: *P* < 0.001, *t* = −18.492) with dangerous than with safe scenes.

These data together suggest that the two dimensions of emotional context (i.e., rich–poor, dangerous–safe) worked independently to affect the neuronal activity and SacRT, and they did so somewhat differently across subjects. Importantly, both the rich and dangerous scenes increased the neuronal activity and decreased SacRT. In fact, there was a significant negative correlation between the neuronal activity and SacRT among all three groups of scenes (poor, rich, dangerous) in all the monkeys (Fig 6C). These data suggest that both rich and dangerous scenes activated amygdala neurons, which in turn led to the facilitation of saccades. Because the context starts working early after a scene appears (but before an object appears), saccades to both good and bad objects were facilitated (Fig 3).

Even though many of these amygdala neurons showed context-dependent activity, their activity was often variable or selective across scenes within the same context (Fig 4). Notably, such variability was different across neurons that are sensitive to the same context in the same monkey (S5A Fig). Presumably, based on the variable variability, amygdala neurons as a population were less variably active in different scenes that belonged to a particular context (S5B Fig). Overall, the scene selectivity tended to be lower in the population activity than in individual neuronal activity, especially before the first saccade (FX period) (S5C Fig) (one-sample *t* test; PA[D/R]: *P* < 0.001, *t* = 5.435; PA[S/R]: *P* = 0.104, *t* = –1.653; PA[S/P]: *P* = 0.986, *t* = –0.017; PI[D/R]: *P* = 0.068, *t* = 1.868; PI[S/R]: *P* = 0.012, *t* = 2.606; PO[S/P]: *P* = 0.023, *t* = –2.345; SO[D/R]: *P* = 0.042, *t* = 2.084; SO[S/R]: *P* < 0.001, *t* = 4.750; SO[S/P]: *P* < 0.001, *t* = 7.091).

Notably, any context is based on the behavioral outcome associated with each environment. What happens if the outcome is changed? To address this question, we let the subjects experience some of the well-learned scenes, but with a different outcome (i.e., no object, no robber) in a nonforaging task and examined some danger-sensitive neurons (S6A Fig). Although the nonforaging task was presented separately from the foraging task, these neurons were still activated by the dangerous scenes immediately after their appearance in the nonforaging task (S6C, S6E and S6G Fig). Moreover, they expressed scene selectivity that was similar to the selectivity in the foraging task. These results suggest that amygdala neurons can be activated automatically when the subject encounters emotional environments that are no longer associated with emotional events. Their activity decreased quickly, however, suggesting that these automatic responses were suppressed by subsequent events in the trial.

**Fig 7:**
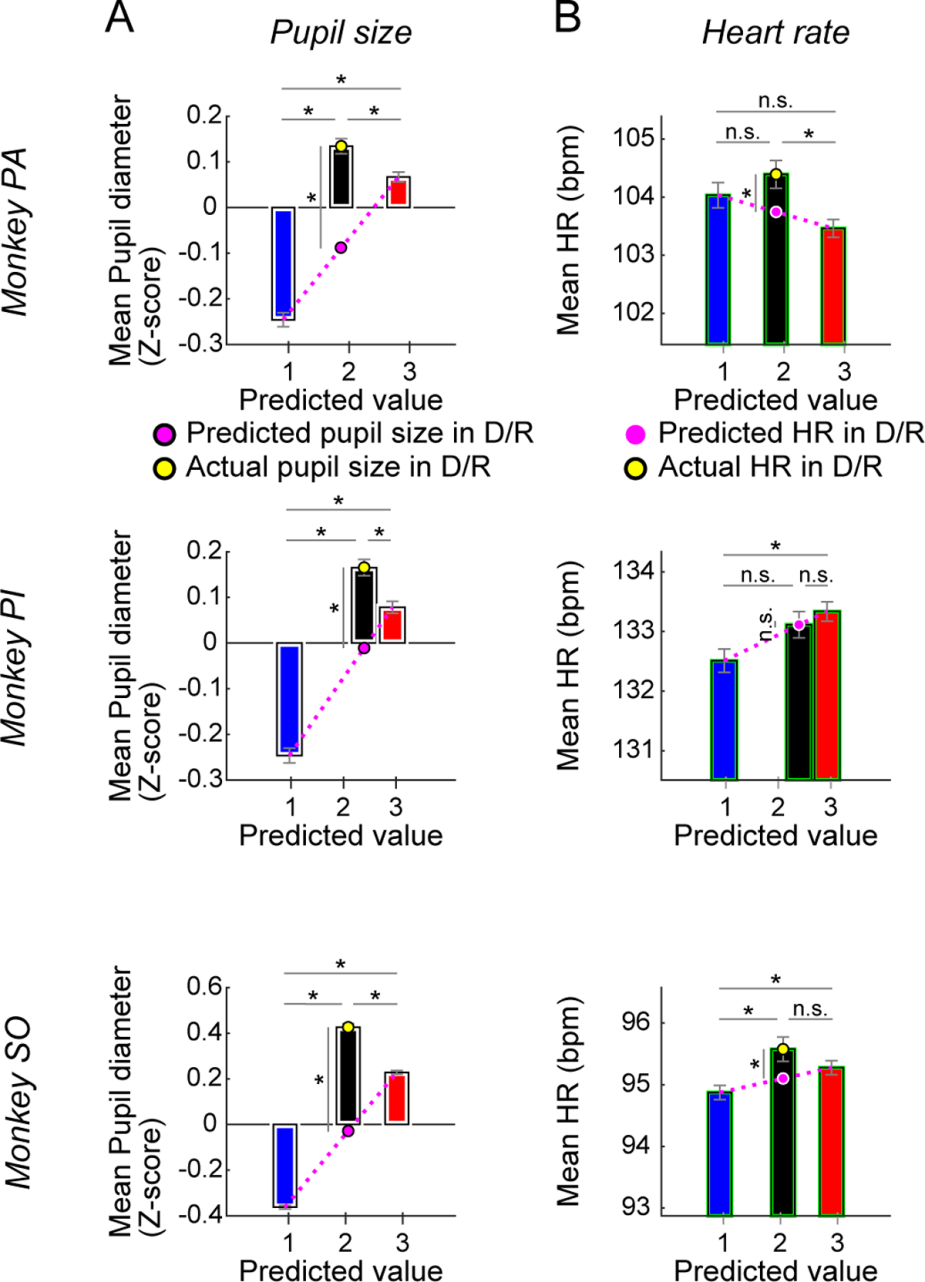
Pupil size and heart rate modulated by two dimensions of environmental context. Pupil size (**A**) and heart rate (**B**) during the fixation period in all three monkeys, shown separately for three groups of environments: S/P, D/R, and S/R. The physiological measures (ordinate) are plotted against the predicted reward value (abscissa), defined as reward volume × success rate (see S2D Fig). Same format as Fig 6A and 6B. Asterisk (*) indicates statistically significant contrasts at *P* < 0.05 (environment context: one-way ANOVA, post hoc: Tukey–Kramer [see S2 Table]; predicted versus actual in D/R: one-sample *t* test, pupil size [PA]: *P* < 0.001, *t* = 14.962, pupil size [PI]: *P* < 0.001, *t* = 10.639, pupil size [SO]: *P* < 0.001, *t* = 39.775; heart rate [PA]: *P* = 0.001, *t* = 2.571; heart rate [PI]: *P* = 0.986, *t* = 0.017; heart rate [SO]: *P* = 0.018, *t* = 2.377). Data used to generate these plots can be found at https://osf.io/2yq8p/?view_only=97c4b290514348bb91cdbb9ec1c85e09. D/R, dangerous and rich; n.s., not significant; S/P, safe and poor; S/R, safe and rich.

Our data so far have shown that the effects of the environment context were somewhat different across subjects. Does this mean that they have different sensitivities to emotion? To address this question, we compared pupil size and heart rate across the three groups of scenes (Fig 7). The pupil size was affected by both dimensions of context (Fig 7A): larger with the rich than the poor scenes and also larger with the dangerous than the safe scenes. This result was seen in all subjects, suggesting that all of the monkeys were sensitive to both richness and danger. The heart rate (Fig 7B) was higher with the rich than the poor scenes in monkeys PI and SO (but not PA) and with the dangerous than safe scenes in monkey PA and SO (but not PI). This result followed the same pattern seen with amygdala neuronal activity and SacRT (Fig 6) and raises the possibility that both heart rate and saccades are modulated by amygdala neurons, based on the environment context.

The mere presence of dangerous scenes affected SacRT to the first object as well as amygdala neuronal activity preceding the saccade in monkeys PA and SO (Fig 6). However, the appearance of an actual robber was a more threatening event. We refer to the presence or absence of a robber object as “object context.” Additionally, some trials with safe scenes contained a distractor object that stayed on the screen but never robbed a good object (S1 Fig). We defined “object context” as the presence or absence of robber or distractor objects. We then examined the effect of object context over and above effects of danger and richness considered thus far (S7 Fig). The data suggest that amygdala neurons could facilitate saccades based on the object context, in addition to the scene context. This is explained in detail in the legend of S7 Fig.

**Fig 8:**
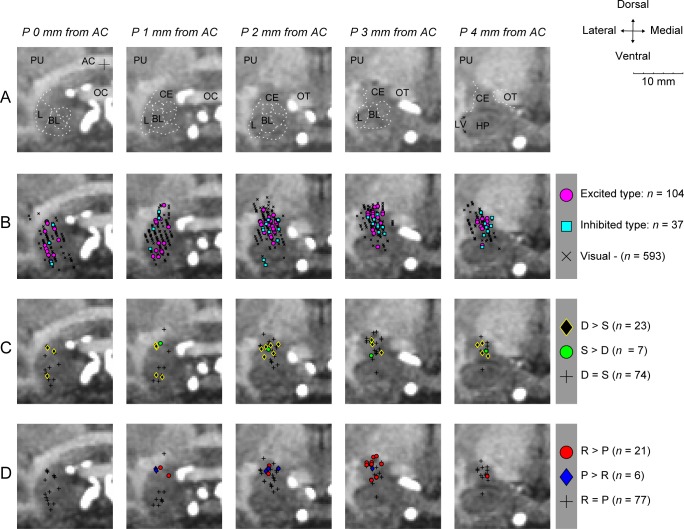
Estimated positions of recorded neurons. Recording sites are shown in five coronal MR images spanning 0–4 mm posterior to the AC. **(A)** Amygdala and surrounding brain areas. **(B–D)** The neurons’ positions are shown, based on different features. **(B)** Neurons excited and inhibited by visual scenes during the fixation period, and neurons showing no response to the scenes (Visual −). **(C)** Excited-type neurons showing significantly different activity between dangerous (D) and safe (S) contexts (D > S; S > D), and others (D = S). **(D)** Excited-type neurons showing significantly different activity between rich (R) and poor (P) contexts (R > P; P > R), and others (R = P). AC, anterior commissure; BL, basolateral complex of the amygdala; CE, central nucleus of the amygdala; D, dangerous; HP, hippocampus; L, lateral nucleus of the amygdala; LV, lateral ventricle; MR, magnetic resonance; OC, optic chiasm; OT, optic tract; P, poor; PU, putamen; R, rich; S, safe.

Finally, we estimated the locations of these neurons by the 3D coordinates of the recording sites that were aligned on magnetic resonance (MR) images (Fig 8). Neurons responding to the visual environments (scenes) were located in various areas in the amygdala, presumably including the central (CE), lateral (L), and basolateral (BL) nuclei (Fig 8B). Neurons that were sensitive to the dangerous–safe dimension of context (D > S; S > D) were located mainly in CE and sparsely in BL and L (Fig 8C). Neurons that were sensitive to the rich–poor dimension of context (R > P; P > R) seem localized in CE (Fig 8D). Compared with neurons in BL and L, neurons in CE had more variable features, including the background firing rate (S8C and S8D Fig) and scene selectivity (S8E and S8F Fig).

## Discussion

### Amygdala neuronal activity correlated with context-based saccades

Goal-directed behavior is affected by various contexts [7]. Furthermore, neurons in various brain areas [4,19–23], including the amygdala [3,24], are sensitive to both target and context information, suggesting that these regions contribute to context-dependent decision-making. However, it was still unclear from previous studies which brain areas process individual contexts, largely because targets and contexts were presented simultaneously. To dissociate these effects, we presented the scene context before the target objects appeared.

This design allowed us to find that many amygdala neurons became active during the appearance of scenes but before objects appeared (Figs 4 and 5). Moreover, the population activity of amygdala neurons was negatively correlated with SacRT in all three monkeys (Fig 6C), suggesting that these neurons facilitate the initiation of saccades. Because the neuronal activity changed before the appearance of an object, it could modulate the whole distribution of SacRTs. This fact could account for the speeding of saccades of all latencies in dangerous or rich contexts (Fig 3A). This parallel shift of the whole SacRT distribution by environment context stands in contrast to the effect of object value on saccades in that good objects cannot attract faster saccades at the shortest latencies (Fig 3B).

We used two dimensions of context (i.e., rich–poor and dangerous–safe) by categorizing many visual scenes. First, in contrast to poor scenes, rich scenes increased amygdala neuronal activity and decreased SacRT in two monkeys (PI and SO) (Fig 6A and 6B). This finding is relevant to studies showing that amygdala neurons encode internally generated reward goals [12,25,26]. Second, the dangerous–safe dimension of context was examined by dangerous scenes where the good object can be removed by a third (“robber”) object (S1B Fig). These dangerous scenes (compared with safe scenes) increased amygdala neuronal activity and decreased SacRT in two monkeys (PA and SO) (Fig 6A and 6B). Importantly, both dimensions of context (rich–poor and dangerous–safe) affected amygdala neuronal activity and SacRT in opposite directions (i.e., increases versus decreases). This suggests that amygdala neurons facilitate saccades based on these two dimensions of environmental context.

Our results are consistent with previous studies showing that the amygdala is involved in gaze orientation and attention [27–31], including a study suggesting that the amygdala enhances attention to visual stimuli associated with rewarding or aversive experiences [32].

### Effects of multiple contexts

These results raise a question: are the two context-based mechanisms operated by the same or by different groups of amygdala neurons? If the same, the two dimensions of context (rich–poor and dangerous–safe) would have similar neuronal–behavioral effects and would do so in all subjects, which was not the case in our study. Thus, two different groups of amygdala neurons may encode the two dimensions of context selectively. The across-subject difference (e.g., monkey PI versus PA) may reflect the biased activation of the amygdala neurons: more activation of the rich–poor neurons in monkey PI and of the dangerous–safe neurons in monkey PA. This speculative mechanism might be related to emotional dimensions [33].

Why, then, did different contexts affect the three monkeys somewhat differently? For example, judging by neuronal responses and saccade metrics, monkey PI seemed insensitive to dangerous scenes (Fig 6A and 6B). However, pupil size was affected by the three groups of scenes (poor, rich, dangerous) similarly in all three monkeys (Fig 7A), suggesting that they shared the same types of emotion or arousal [34–36]. These data suggest that a change in the emotional state started affecting the amygdala-saccade mechanism in different time courses across subjects: before the robber object appeared in monkeys PA and SO (Fig 6A and 6B) and after the robber object appeared in monkey PI (S7B Fig). In contrast with pupil size effects, heart rate effects matched the pattern of changes seen in the amygdala recordings and saccade metrics (Figs 7B, 6A and 6B).

We also found that both amygdala neuronal activity and SacRT were affected by the presence of nonthreatening distractor objects that never robbed the reward (S7 Fig). Because the success rate (i.e., probability of rewarded trials) was not significantly different between the distractor present or absent trials, the change in SacRT is unlikely to be caused by the dangerous context. Instead, it may be related to a higher demand of attention when two objects are present simultaneously [37].

### Neural circuit model for target choice modulated by emotional contexts

**Fig 9:**
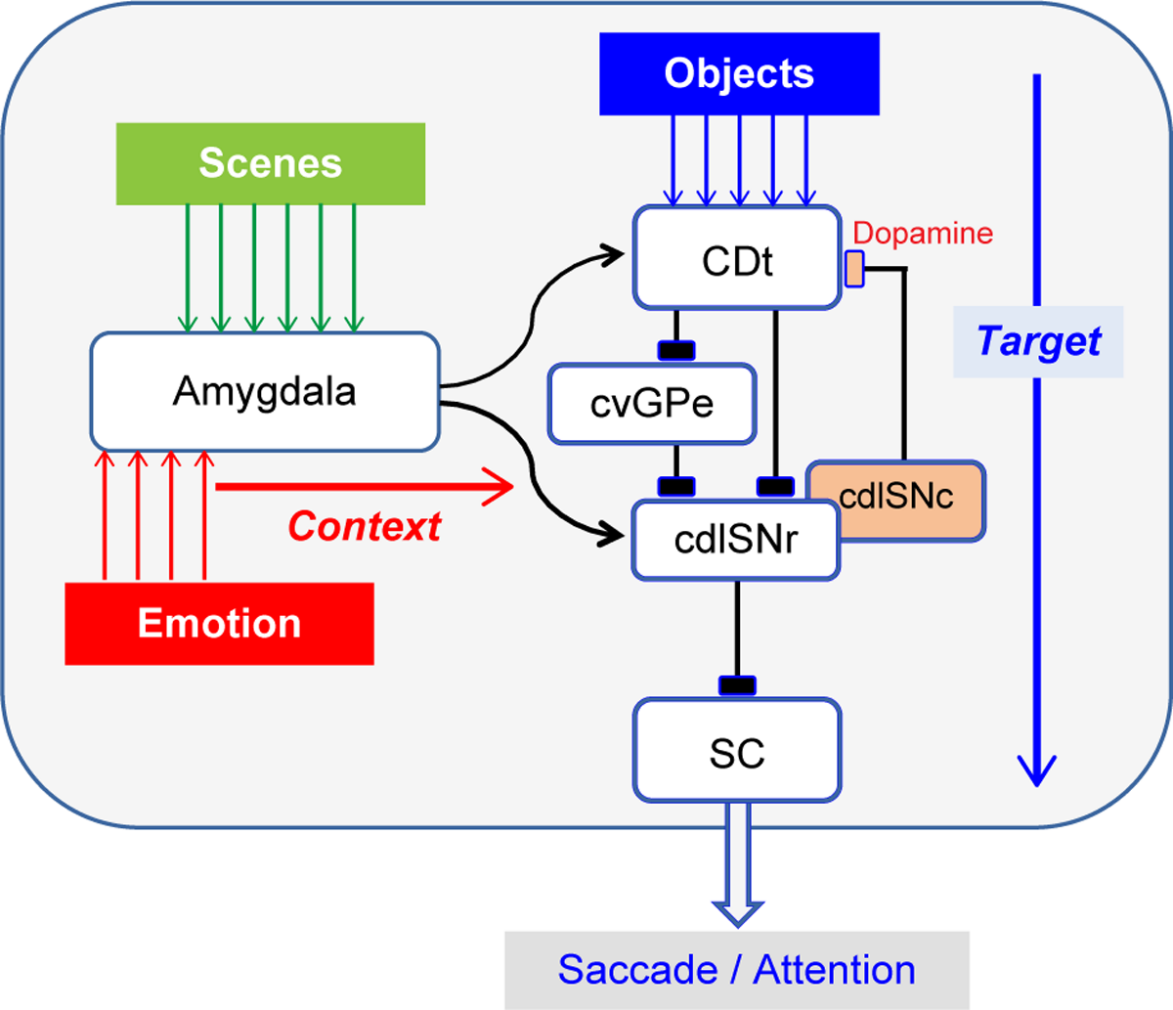
How emotional–environmental contexts affect saccadic eye movement—hypothetical neural circuits. cdlSNr, caudal-dorsal-lateral part of the substantia nigra pars reticulata; cdlSNc, caudal-dorsal-lateral part of the substantia nigra pars compacta; CDt, caudate nucleus; cvGPe, caudal-ventral part of the globus pallidus externus; SC, superior colliculus.

Based on these results, we propose a scheme for the context-target interaction (Fig 9). During the foraging task used in this study, a particular circuit originating from the tail of the caudate nucleus (CDt) is likely to control the targeting of saccades. Its final output station is the caudal-dorsal-lateral part of the substantia nigra pars reticulata (cdlSNr), where neurons discriminate between good and bad objects about 100 ms after object appearance in the contralateral-peripheral position and send the information to the SC [38]. Therefore, the CDt-cdlSNr-SC circuit may cause the object-based change in SacRT in our foraging task (Fig 3B). Importantly, this mechanism works regardless of the context.

This basal ganglia circuit may be modified by the inputs from the amygdala [39–43], which convey multiple dimensions of context information. The caudal-ventral part of the striatum (including CDt) receives inputs from the basal nucleus of the amygdala (BA) [39,43], which in turn can facilitate corticostriatal synaptic plasticity [44]. In addition, the substantia nigra pars compacta (SNc) and pars reticulata (SNr, including cdlSNr) receives inputs from the CE [40–42]. Notably, most neurons sensitive to the scene-based context were located in or close to CE (Fig 8) [45]. In this sense, the CE–SN connection [46] might play a major role.

In our foraging task the performance of the monkeys was extraordinary in several aspects, specifically fast learning, high-capacity memory, and fast saccadic reactions. The SacRTs were largely in the range of express saccades [47,48], which are controlled directly by SC [49,50]. This SC mechanism may be promoted by amygdala neurons that were sensitive to the emotional contexts (i.e., dangerous, safe, rich, poor). Such emotional signals may be used for defensive behavior through the SNr–SC circuit [51], consistent with our scheme (Fig 9). This mechanism might work commonly in real-world contexts [52,53].

### Categorization of scenes into contexts

How, then, does the amygdala obtain information? The subjects experienced many scene images, each of which acted as an environment that contained two or three objects. We therefore speculate that the amygdala receives environmental information from scene-sensitive neurons (Fig 9). Indeed, the parahippocampal area contains scene-sensitive neurons [8,54] and also projects to the amygdala [55]. Notably, some amygdala neurons responded to the scenes variably, even among scenes that belonged to the same context (S5 Fig). These findings suggest that each amygdala neuron intrinsically receives inputs from a variable assortment of scene-sensitive neurons. As a result, some of the amygdala neurons would have limited information about the contexts. As a population, however, because the within-context variability is lower, the across-context differences are signaled clearly (S5 Fig). An equivalent process of categorization is present in the CDt-cdlSNr-SC circuit, through which target context could be established [56].

However, the scenes themselves may have no information about context. Context is created by learning—that is, by experiencing various events repeatedly in each scene, such as the occurrence of robbers or big rewards [3, 7]. Because the contexts in our foraging task were emotional (i.e., dangerous, safe, rich, poor) [16], the information of each scene must be modified by a separate source of information related to the emotional events (Fig 9). This may be controlled by various inputs from emotion-sensitive brain areas [57,58] and neuromodulatory neurons, including dopamine, norepinephrine, serotonin, oxytocin, and acetylcholine [30,59]. Thus, the cortico-amygdala connection may be modulated by the emotional inputs, for example, another person's direct gaze [60].

### Social–emotional roles of saccade

If the amygdala mechanism (Fig 9) is disrupted, the automatic saccades to target objects would be suppressed in some contexts. This actually occurs in human subjects with amygdala lesions [61]. When facing other humans, they rarely look at their eyes, unlike control subjects. This appears to be dependent on social context (in which fearful emotions might be evoked), because such saccade suppression can be eliminated when only a small region of the face is made visible [62]. Similar changes in social–emotional saccades occur in human subjects with amygdala dysfunctions (e.g., autism) [63,64]. These results suggest that the amygdala mechanism on saccade attention is important personally and socially.

We have suggested that the amygdala modulates goal-directed behavior based on emotional contexts. However, different kinds of context are likely controlled by different brain areas [65,66]. For example, something unexpected often suppresses ongoing behavior, which may be controlled by dorsomedial cortical areas [67,68]. Overall, many brain areas may modulate behavior based on specific or integrated contexts [69,70].

## Materials and methods

### Ethics statement

All animal care and experimental procedures were approved by the National Eye Institute Animal Care and Use Committee (proposal number: NEI-622) and complied with the Public Health Service Policy on the humane care and use of laboratory animals. Anesthesia was induced with ketamine and diazepam, after which animals were intubated and then maintained with isoflurane. Animals had respiratory, blood pressure, and electrocardiogram monitors and were placed on a heating pad throughout the period of anesthesia.

### General procedures

Three adult male monkeys (rhesus macaque), PA (9.0 kg, 9 y old), PI (13.0 kg, 7 y old), and SO (9.0 kg, 6 y old), were used for behavioral testing and neuronal recording.

We implanted a plastic head holder and a plastic recording chamber to the skull under general anesthesia and sterile surgical conditions. One search coil was surgically implanted under the conjunctiva of an eye in each monkey to record eye movements. After the monkeys fully recovered from surgery, we started training them on the foraging task.

### Foraging task

Behavioral tasks were controlled by a custom neural-recording and behavior-controlling system (Blip; available at http://www.robilis.com/blip/). The monkeys sat in a primate chair facing a fronto-parallel screen in a sound-attenuated and electrically shielded room. Visual stimuli were rear-projected on the screen by a digital light processing projector (PJD8353s, ViewSonic). Eye position was sampled at 1 kHz using the scleral search coil (monkey PA) or a video-based eye tracker (EyeLink 1000 Plus, SR Research; monkeys PI and SO).

We devised a foraging task in which the monkeys viewed many scenes presented in randomized order across different trials (Fig 1C). Each scene contained at least two objects with different features (S1 Fig). Objects #1 and #2 were responsive to the subject’s choice (i.e., sustained gaze). Saccades to object #1 gave the subject a reward (called “good” object), whereas saccades to object #2 gave no reward (called “bad” object). Thus, the subject’s goal was to make saccades to object #1 but not object #2. Such objects were present in all scenes, although they were visually different. Some scenes also contained the third object (object #3, “robber” or “distractor”). Based on the effects of these objects, the scenes were classified into several groups, as described below.

We will first describe the physical features of the scene and object (Fig 1). The scene was a large visual image (circular, radius: 25 deg) that was projected onto a screen in front of the subject (distance: 45 cm). The scenes were grayscale images derived from Google Earth imagery (https://www.google.com/earth/). Example scene images in Figs 1, 2A, S2A Fig, S1 and S2 Movies were derived from OpenAerialMap (https://openaerialmap.org). The objects were computer-generated fractals with multiple colors (radius about 5 deg). They were small enough to be presented at different positions in the scene (Fig 1). Because it was unlikely that the subject had seen any of the objects or scenes before the experiment, we could control the levels of both object–reward association learning and scene–context association learning. Furthermore, we could generate an infinite number of novel objects and novel scene images. These features allowed us to repeat these association learnings with fresh visual stimuli.

To examine the behavioral and neuronal encoding of scenes (environments), both the learning (of the meanings of objects and scenes) and testing (of the monkey’s behavior and of the activity of the amygdala neurons) were done in the same task procedure (Figs 1 and S1). After the ITI (4–8 s), a scene appeared suddenly and the subject was allowed to view the scene freely for 1,080 ms (FV period). Then, a fixation point (FP) appeared at the center. If the subject held its gaze on the FP for 780 ms (FX period), an object appeared at the same time as the FP disappeared. The object appearance was random and unpredictable in two ways: (1) sequence: two or three objects contained in the scene appeared randomly in sequence and (2) position: the objects appeared randomly at one of eight positions (eccentricity: 15 deg; angle: in steps of 45 deg from straight up).

A response to object #1 (good) or #2 (bad) occurred if the subject made saccade to it within 1,000 ms and kept fixating on it (>400 ms). This action was followed by a reward with the good object and no reward with the bad object, either of which ended the trial. Depending on the scene, the size of reward was either big (0.3 mL) or small (0.1 mL). To gain a reward the subject needed to refrain from responding to the bad object so as to wait for the good object. There were two ways to perform “no action”: (1) no saccade to the bad object or (2) saccade to the bad object followed quickly (within 400 ms) by a second saccade away from the bad object. Both subjects performed the latter no-action behaviors more commonly. After the no-action, the FP reappeared and the object presentation cycle was repeated, with the possibility that the good object might appear (S1 Fig).

Some scenes were designated as “dangerous” contexts and could feature the appearance of a third type of object (called the “robber” object). After appearing, it remained in place (irrespective of the subject’s eye movements) until either the good or the bad object appeared. If the bad object appeared, the robber object did nothing and the subject simply needed to make no action before FP reappeared. If the good object appeared, the robber was programmed to jump across the screen and interrupt the object with timing designed to race against the monkey’s saccade to the good target. On trials for which the robber’s jump preceded the monkey’s response, the robber “stole” the reward that would otherwise follow a correct saccade to the good object. Stolen trials occurred if the subject’s saccade was delayed (reaction time: >90–120 ms for monkey PA, >100–180 ms for monkey PI, > 95–125 ms for monkey SO; the threshold was random across trials between these numbers). Otherwise, the subject obtained the reward (S1 Fig). If the subject failed (i.e., the robber beat the saccade), the same trial was repeated in the same sequence until a successful trial (i.e., the saccade beat the robber), but these repeated trials were not included in behavioral and neuronal analysis. To encourage the subject, the criterion reaction time was incremented by 10 ms after each failure. There was another effect of the robber object in some scenes (#170–173, #190–193, #240–243, 280–283, shown in Fig 1C): on trials in which the subject failed (robber wins), an air puff was delivered to the subject’s face. A third object also appeared in some scenes that were designated as “safe” scenes. This object #3 was called the “distractor” object and, like the robber, lingered on the screen but never attacked the good object (S1 Fig).

Monkeys PA and PI both learned 56 scenes and 140 objects, and monkey SO learned 32 scenes and 68 objects. In each experimental session, many of the scenes (i.e., 32 scenes for each subject) were presented randomly.

These combinations of scenes and objects together constituted two dimensions of context.

(1) Dangerous (D) versus safe (S):
  Dangerous: the robber object is present and might appear but does not do so on every trial.
  Safe: the robber object is absent.
(2) Rich (R) versus poor (P):
  Rich: the good object is associated with a big reward (0.3 mL).
  Poor: the good object is associated with a small reward (0.1 mL).
  By combining the context dimensions in a 2 × 2 matrix, we created environments matching three of the possible combinations (Fig 1C):
  (1) dangerous and rich (D/R)
  (2) safe and rich (S/R)
  (3) safe and poor (S/P)
  For practical purposes, the fourth (D/P) combination was not used. Pairwise contrasts in behavioral or neuronal activity between groups indicated a context effect, as follows:
  (1) D/R versus S/R: dangerous–safe context.
  (2) S/R versus S/P: rich–poor context.

### Nonforaging task

Some neurons were tested in this task to investigate whether recorded neurons could discriminate the contexts or environments absent the usual the outcomes (S6A Fig). At the beginning of the trial, the monkeys had to keep fixating on FP until FP disappeared (700–1,200 ms). Then, a scene image was presented for 2,000 ms at the center of the screen and the subject was allowed to view the scene freely. After that, the scene image disappeared and the FP appeared at a random peripheral position (10 degrees from center). If the monkey continued fixating on the FP until it disappeared (700–1,200 ms), a reward was delivered.

### Electrophysiology

Based on a stereotaxic atlas (Saleem and Logothetis, 2007), we implanted a rectangular chamber targeting the amygdala. The chamber was tilted laterally by 20 degrees for monkeys PA and PI and tilted anteriorly by 15 degrees for monkey SO. MR images (4.7 T, Bruker) were then obtained along the direction of the recording chamber, which was visualized with gadolinium that filled grid holes and the space outside the grid and inside the chamber. Single-unit recording was performed using glass-coated electrodes (Alpha-Omega). The electrode was inserted into the brain through a stainless steel guide tube and advanced by an oil-driven micromanipulator (MO-97A, Narishige). The recording sites were determined by using a grid system that allowed recordings at 1-mm intervals in x- and y-directions, orthogonal to the guide tube. The input from the electrode was amplified with a band-pass filter (0.2–10 kHz; BAK). Neuronal spikes were isolated online using custom software implementing a voltage and time window discriminator (Blip). To find visually responsive amygdala neurons, we let the monkey continue to perform the foraging task and checked responses to scene images and object images. We examined any neuron systematically if it responded to at least one scene image (*n* = 54 in monkey PA, *n* = 50 in monkey PI, and *n* = 54 in monkey SO; see S8B Fig) using the foraging task.

### Quantification and statistical analysis

#### Behavior analysis

In order to study the pure effect of the environment (scene) on the targeting behavior, we analyzed the reaction time of the first saccade to an object (either good or bad object) in each trial. Up to this point, everything was the same across trials, except for the scene (see Fig 1A and 1B). For the object context, we analyzed the second or later saccades, both before and after distractor/robber was presented.

To evaluate the effects of the scene–context and object–reward association learning, we measured two parameters: (1) the probability of choosing good objects (S2B Fig) and (2) the SacRT (S2C Fig). The choice probability was calculated as follows: “Choice probability = N_good_ / (N_good_ + N_bad_),” where N_good_ and N_bad_ are the number of trials of choosing the good object and the bad object, respectively. SacRT was measured as the time between the offset of the FP (simultaneous with the onset of the object) and the onset of the saccade to the fractal object. Saccades were detected when the peak velocity of the polar component exceeded 300 degrees/s. Saccade onset time was defined as the time point preceding the detected saccade at which the velocity exceeded 30 degrees/s.

As described in Figs 2, 3, and 6, SacRT was measured only for the saccade to the first object (either good or bad object) in each trial. SacRT for trials in which the robber or distractor objects appeared was analyzed in S7A Fig. Repeat trials showing the same scene after errors, failure trials, or saccades to wrong directions were excluded from analysis. To compare the SacRTs in different contexts (Fig 6), the mean SacRTs in these contexts were calculated and compared with one-way ANOVAs (three groups [D/R, S/R, S/P], post hoc: Tukey–Kramer).

To compare SacRT between different contexts and objects, the cumulative distributions of SacRTs were plotted on a probit scale with a reciprocal time axis (reciprobit plot, Figs 2 and 3) [18]. In this procedure a cumulative Gaussian distribution was transformed to a straight line as reciprobit plot. Variation in the mean rate of the distribution leads to horizontal, self-parallel translation of the reciprobit plot [18]. Altering the variance of a distribution rotates the plot around its median.

Additionally, we examined pupil size and heart rate in the foraging task. Pupillary changes were recorded by using video-based eye tracker (EyeLink 1000 Plus, SR Research). Heart rates were measured by using a pulse sensor (World Famous Electronics llc) at the ear. Outliers related to eyelid closure or loss of the eye-tracking signals were detected and removed. Mean pupil diameter in each trial was calculated during 500 ms before target onset, when gaze was fixed (FX period). Heart rate was calculated from recent interpulse interval before target onset in the FX period. These values were converted to z-scores across trials.

#### Neuronal activity analysis

A main purpose of this study was to examine the pure effect of the environment (scene) on neuronal activity. For this purpose, we focused on the neuronal activity in the early stage of each trial (i.e., after the scene appeared and before the first object appeared) (see Fig 2A). Immediately after this epoch, the first saccade to the object occurred, which we analyzed specifically (see above, Behavior analysis). This early stage contained two periods: FV period (scene onset to FX start timing, around 1,100 ms) and FX period (FX start to object onset timing, around 800 ms). We analyzed neuronal data for these periods separately as well as in combination.

First, we classified neurons into either excited type or inhibited type (Fig 5). This was determined by the difference in the firing rate between FX period and baseline period (500–0 ms before scene presentation) in one of the environment groups (D/R, S/R, or S/P) that affected the neuron most strongly. If the firing rates were significantly larger or smaller than the baseline firing rates (*P* < 0.05, Wilcoxon rank-sum test), the neuron was defined as either excited or inhibited type, respectively. The excited-type neurons were used to calculate population neuronal activities for environment contexts.

We measured the latency of the neuronal response to the scene in general (S4 Fig). It was calculated for the excited-type neurons. At each time point after scene onset, the averaged discharge rate was calculated during the 35-ms period before that time point (period A) and during the preceding 200-ms period (i.e., the 235–35 ms period before the time point) (period B). Then, the average discharge rate during period A was compared with that during period B (student *t* test). If the averaged discharge rate was significantly different between period A and period B (*P* < 0.05), that time point was regarded as a time of significant difference. This procedure was repeated by shifting the time point in 1-ms steps after scene onset. If, over 30 consecutive 1-ms steps, the first and at least 26 subsequent showed significant differences, that first time point was defined as the latency of responses. If the latency was not detected until object onset, period B was fixed during the 200-ms period before the scene onset.

We then examined whether the neurons discriminated between contexts in the two dimensions (dangerous versus safe, rich versus poor) and measured the latency of the discrimination (S4 Fig). First, we measured the discrimination latency for neurons during the combined test window (FV–FX period). At each time point after the latency of the scene response, the neurons’ averaged discharge rate was calculated during 30 ms before the time point in each context. If, over 30 consecutive 1-ms steps, the first and at least 26 subsequent showed significant differences, that first time point was defined as the onset of discrimination signaling. Then, we measured the magnitude of the neuron’s response in each context by counting the numbers of spikes within each of the two test windows (FV and FX periods) in individual trials. The neuronal discrimination score was calculated as ΔFR based on differences of response magnitudes of the amygdala neurons between two groups of environments (D/R versus S/R, S/R versus S/P; see Fig 4), each of which contrasts a particular context dimension (dangerous–safe, rich–poor). The statistical significance of neuronal discrimination was tested using one-way ANOVA and Tukey–Kramer post hoc test in each of the two test windows (FV and FX periods) (Figs 4 and S3). To estimate the selectivity of neuronal responses to scenes in each context, the selectivity index (SI) was calculated based on the mean FR in each scene.

SI = 1 − (FRall / FRm × NS),

FRall: the total of FR in all scenes in the context,
FRm: the largest FR among tested scenes,
NS: number of tested scenes.

We compared the responses of amygdala neurons to different contexts as well as individual scenes (Figs 6 and S5). For each neuron, we calculated the mean firing rate to each scene in each neuron, which was converted to normalized z-scores: (FR_i_ − FR_b_) / SD, (FR_i_: mean firing rate during the test window, FR_b_: baseline firing rate, SD: standard deviation of mean firing rates for all scenes). We then averaged z-scores of all scene-responsive neurons for each context of scenes (e.g., Fig 6A) and for each scene (e.g., S6 Fig). To examine the significance of context discrimination we used one-way ANOVA (three groups [D/R, S/R, S/P], post hoc: Tukey–Kramer).

The dangerous–safe dimension of context was estimated basically by the comparison between the two groups of scenes (D/R versus S/R) because the predicted reward amount was the same (big). However, the average reward amount was smaller in D/R than S/R because the reward was sometimes removed by the robber object. Therefore, we also used another method based on the predicted reward values for neuronal activity (Fig 6). The actual neuronal activity in D/R was compared with the predicted neuronal activity (PN) based on the predicted reward value in D/R.

PN = FRp + (FRr − FRp) × (PVd − PVp) / (PVr − PVp),

FRr/FRp: neuronal activity (mean firing rate) in S/R or S/P,
PVd/PVr/PVp: predicted reward value in D/R, S/R, or S/P.

The predicted reward value was calculated by a following equation: PV = R × S × 10 (R: reward amount when the monkey chose the good object [0.3 mL in rich and 0.1 mL in poor environments], S: rate of successful [rewarded] trials).

The same method was used for pupil size and heart rate (Fig 7).

The effect of object context on neuronal activity was evaluated based on the effects of the robber and distractor objects (S7 Fig). We compared the neuronal activity in the FX period between the two object contexts: robber/distractor (−) (before the robber/distractor object appeared) and robber/distractor (+) (after the robber/distractor object appeared). We analyzed neuronal and SacRT data in these object contexts separately as well as in combination.

## Supporting information

S1 Fig (Related to Fig 1). Foraging task procedure. (**A**) The sequence of events on trials with additional (robber or distractor) object. An environment (scene) appears first, which contains two or three objects with meanings shown in (**B**). After gaze fixation at the center, one of the objects appears at a random position. A saccade to the good object followed by sustained gaze terminates the trial with delivery of reward. The amount of reward is either big or small depending on the scene, creating the rich versus poor dimension of context. Saccades to the bad object followed by sustained fixation terminate the trial with no reward. The subject thus learns to avoid the bad object by either withholding a saccade or leaving the bad object quickly, after which the fixation point reappears. Another object, either robber or distractor, remains for a while, irrespective of the animal’s behavior. The distractor simply remains on the screen, whereas the robber jumps to the good object (if present) and precludes reward delivery if it beats the monkey’s saccade (**C**, see S2 Movie). The presence or absence of the robber determines the dangerous versus safe dimension of context. Both dimensions of context were examined by varying the dimension of interest, while the orthogonal dimension was constant. See Fig 1 and Materials and methods.
(TIF)

S2 Fig Fast learning of environmental contexts and object values. (**A**) Learning across 4-d sessions in monkey PI during the foraging task (4 scenes with 8 objects). Before this experiment the subject had learned the task rule completely, but all scenes and objects were completely new on Day 1. Each session consisted of 52 trials (13 trials for each scene). In the early stage of learning on Day 1, the subject’s choice was sometimes wrong (i.e., bad object) or invalid (i.e., fixation break) but became nearly perfect toward the end of the session. The speed of learning is shown as the change in the correct response rate. The good performance was well retained across days. These example scene images were derived from OpenAerialMap (https://openaerialmap.org). (**B–D**) Learning in three monkeys (PA, PI, SO). (**B**) Time course of object choice learning for many scenes (56 scenes for monkey PA and PI, 32 scenes for monkey SO). (**C**) Change in saccade reaction time to the good object across learning. For each subject, data are shown separately for three scene types: D/R, S/R, and S/P. Trial number was measured for individual scenes, not the subject’s task career. (**D**) Performance after learning (>200 trials for each scene) in the three groups of scenes. “Failure” indicates the D/R context trials in which the robber beat the monkey’s saccade. Dangerous trials featured the appearance of the robber; those that did are indicated in light gray. D/R, dangerous and rich; S/P, safe and poor; S/R, safe and rich.
(TIF)

S3 Fig (Related to Fig 4). (**A**) Neuron in monkey PI (#234) that was selectively active in the rich context during the fixation period (S/R > S/P). (**B**) Neuron in monkey SO (#339) that was active in both the dangerous and rich contexts during the fixation period (D/R > S/R and S/R > S/P). The same format as in Fig 4. S/P, safe and poor; S/R, safe and rich.
(TIF)

S4 Fig Neuronal response latency and differentiation time. Latencies of neuronal responses to visual scenes. (**A**) General latency: time when the neuronal activity (PA, *n* = 33; PI, *n* = 32; SO, *n* = 39) changed significantly after the onset of any of the tested scenes. (**B–C**) Context-discrimination latency: time when the neuronal activity changed significantly between dangerous and safe scenes (**B**, PA, *n* = 27; PI, *n* = 20; SO, *n* = 18) and between rich and poor scenes (**C**, PA, *n* = 25; PI, *n* = 23; SO, *n* = 27). Data are based on excited-type neurons (see S4 Fig) in monkey PA, PI, SO, and all.
(TIF)

S5 Fig (Related to Fig 4D). Scene selectivity of individual and population neuronal activity. (**A**) Scene selectivity of two example neurons in monkey PA. Left: the neuron’s activities in the three groups of scenes: D/R, S/R, S/P. Right: the neuron’s responses to individual scenes in the dangerous (D/R) context (in the fixation period). Same format as in Fig 4B and 4D. Shaded gray area indicates the tested scenes for each neuron. Scene selectivity (SI): 0.473 (#73), 0.775 (#83). (**B**) Scene selectivity of the population neuronal activity (excited-type neurons, *n* = 33) in D/R context in monkey PA (SI: 0.234). Same format as in (A). (**C**) SIs in individual neuronal activity (small dots) and the population neuronal activity (gray squares) in three contexts in each monkey (PA, PI, SO). Mean SI: 0.386 (PA[D/R]), 0.404 (PA[S/R]), 0.345 (PA[S/P]), 0.299 (PI[D/R]), 0.298 (PI[S/R]), 0.264 (PI[S/P]), 0.245 (SO[D/R]), 0.305 (SO[S/R]), 0.311 (SO[S/P]). Population SI: 0.234 (PA[D/R]), 0.444 (PA[S/R]), 0.346 (PA[S/P]), 0.261 (PI[D/R]), 0.248 (PI[S/R]), 0.308 (PI[S/P]), 0.203 (SO[D/R]), 0.180 (SO[S/R]), 0.145 (SO[S/P]). On the right, the probabilistic distribution of SIs is shown by kernel density estimation (width = 0.03) for each context and in each monkey. D/R, dangerous and rich; S/P, safe and poor; S/R, safe and rich; SI, selectivity index.
(TIF)

S6 Fig Effect of environment outcome change on amygdala neuronal activity. (**A**) Nonforaging task. Reward was delivered if the subject kept gaze on the fixation point that moved from the center to a random peripheral position. Before the position change, a well-learned scene was presented for 2,000 ms, during which free viewing was allowed. Across trials, different scenes appeared randomly, but no object (good, bad, robber, distractor) appeared inside the scene. (**B–G**) Activity of three neurons in monkey PA during the foraging task (**B, D, F**) and nonforaging task (**C, E, G**). These tasks were presented as separate blocks of trials. Their responses to individual scenes are also shown. The FV and FX period in the foraging task was combined. FV, free-viewing; FX, fixation.
(TIF)

S7 Fig Effects of dangerous or distracting objects on amygdala neuronal activity and SacRT. **(A)** Distributions of the SacRT to good objects in three monkeys (PA, PI, SO) in three groups of scenes: S/P, D/R, S/R. In each panel, data from two groups of object context are superimposed: Robber(+) and Robber(−) in D/R, and Distractor(+) and Distractor(−) in S/R and S/P. SacRT distribution is shown using reciprobit plot. (**B**) Relation between the neuronal activity (abscissa) and SacRT (ordinate) in two dimensions of context: environment contexts (D/R, S/R, S/P) and object context (Robber/Distractor[+], Robber/Distractor[−]). The object contexts shown here correspond to data shown in (A). (**C**) Relation between the neuronal activity (abscissa) and SacRT (ordinate) for individual scenes. Some scenes provide two data points: Robber/Distractor(+) and Robber/Distractor(−). Findings are presented in further detail below. In the dangerous scene (D/R), SacRT was shorter when a robber object was present (Robber+) than when absent (Robber−) (**B**). The distribution of SacRT became curved, indicating that the saccade preparation process became non-Gaussian by including extremely short SacRTs (**A**, D/R). Notably, these effects occurred in the all monkeys. Taken together with data shown in **B**, these results show that all monkeys were sensitive to danger, in both scene and object (i.e., robber present or absent) contexts. Monkey PI was primarily sensitive to the object context (faster when a robber object was present), in tandem with amygdala neurons tending to be more active on Robber(+) trials than on Robber(−) (**B**), although the statistical significance was shown only in monkey SO. Results of two-way ANOVA tests with environments and object+/− with Tukey–Kramer post hoc tests were as follows. Monkey PA: F[2, 1882] = 0.025, *P* = 0.975, post hoc, *P* = 0.811. Monkey PI: F[2, 1773] = 1.149, *P* = 0.317, post hoc, *P* = 0.882. Monkey SO: F[2, 1958] = 0.425, *P* = 0.654, post hoc, *P* = 0.0422. In the safe scenes (S/R, S/P), SacRT was shorter when a distractor object was present than when absent in monkeys PA and SO (**B**). Because the success rate (i.e., probability of rewarded trials) was not significantly different between the distractor present and distracter absent trials (PA[S/R]: chi-squared = 0.286, *P* = 0.593; PA[S/P]: chi-squared = 0.225, *P* = 0.636; PI[S/R]: chi-squared = 0.205, *P* = 0.651; PI[S/P]: chi-squared = 0.020, *P* = 0.887; SO[S/R] chi-squared = 3.274, *P* = 0.070; SO[S/P]: chi-squared = 2.954, *P* = 0.086), the change in SacRT is unlikely to be caused by the dangerous context. Instead, it may be related to a higher demand of attention when two objects are present simultaneously [19]. Amygdala neurons tended to be more active when a distractor object was present (Distractor+) than when absent (Distractor−) in monkeys PA and SO (**B**), although this trend did not reach statistical significance (two-way ANOVA with environments and object+/−, post hoc: Tukey–Kramer; PA[S/R]: *P* = 0.965; PA[S/P]: *P* = 0.890; PI[S/R]: *P* = 0.930; PI[S/P]: *P* = 0.998; SO[S/R]: *P* = 0.483; SO[S/P]: *P* = 0.716). The integration of neuron-behavior data in the object context (i.e., presence and absence) (**C**) shows that the activity of amygdala neurons was significantly correlated with SacRT in monkeys PA and SO (PA: *r* = −0.288, *P* = 0.005; PI: *r* = 0.022, *P* = 0.833; SO: *r* = −0.660, *P* < 0.001). These data suggest that amygdala neurons could facilitate saccades based on the object context, in addition to the scene context. D/R, dangerous and rich; S/P, safe and poor; S/R, safe and rich; SacRT, saccade reaction time.
(TIF)

S8 Fig (Related to Fig 8). Recording sites. (**A**) Amygdala and surrounding brain areas. Same format as in Fig 8A. A horizontal black line indicates the dorsoventral border of neurons based on their background firing rate (data shown in D). (**B**) Neurons with visual responses (Visual +) and with no visual responses (Visual −), shown separately for three monkeys. (**C**) Visual scene-sensitive neurons (excited type) are classified based on their background FRs. (**D)** Background FRs of individual neurons (abscissa) plotted against their recorded depths from the AC (ordinate). The horizontal line (i.e., 8 mm below AC) indicates the dorsoventral border, by which the variance of Background FR was maximally higher in the dorsal area than the ventral area. This was determined by the lowest *P* value (two-sample F-test) while moving the border line (0.2 mm step) (as shown in colored bars on the right) (F[108, 48] = 3.363, *P* < 0.001, dorsal [355.856], ventral [105.812]). Note that the border line roughly corresponds to the border between CE and BL/L. (**E-F**) SIs of individual neurons (abscissa) plotted against their recorded depths (ordinate) during the free-viewing period (**E**) and the fixation period (**F**). With the border based on Background FR (D), the variance of SIs was significantly higher in the dorsal area than the ventral areas (SI in free-viewing: F[324,146] = 1.958, *P* < 0.001, dorsal [0.030], ventral [0.015]; SI in fixation: F[326,146] = 1.476, *P* = 0.008, dorsal [0.033], ventral [0.023]). AC, anterior commissure; BL/L, basolateral complex and lateral nucleus of the amygdala; CE, central nucleus of the amygdala; FR, firing rate; SI, Selectivity index.
(TIF)

S1 Movie (Related to Fig 1 and S1 Fig). Monkey’s gaze positions during performance of the foraging task (safe and rich context). Yellow dots show monkey’s gaze positions. The example scene image was derived from OpenAerialMap (https://openaerialmap.org).
(MOV)

S2 Movie (Related to Fig 1 and S1 Fig). Monkey’s gaze positions during performance of the foraging task (dangerous and rich context). Same format as S1 Movie. The example scene image was derived from OpenAerialMap (https://openaerialmap.org).
(MOV)

S1 Table (Related to Fig 6). Statistical values for the neuronal activity and SacRT. SacRT, saccade reaction time.
(XLSX)

S2 Table (Related to Fig 7). Statistical values for the pupil size and HR. HR, heart rate.
(XLSX)

## Abbreviations

BA: basal nucleus of the amygdala
BL: basolateral complex of the amygdala
BL/L: basolateral complex and lateral nucleus of the amygdala
cdlSNc: caudal-dorsal-lateral part of the substantia nigra pars compacta
cdlSNr: caudal-dorsal-lateral part of the substantia nigra pars reticulata
CDt: tail of the caudate nucleus
CE: central nucleus of the amygdala
cvGPe: caudal- ventral part of the globus pallidus externus
D/P: dangerous and poor
D/R: dangerous and rich
FP: fixation point
FV: free-viewing
FX: fixation
L: lateral nucleus of the amygdala
LV: lateral ventricle
MR: magnetic resonance
PN: predicted neuronal activity
S/P: safe and poor
S/R: safe and rich
SacRT: saccade reaction time
SC: superior colliculus
SI: selectivity index
SN: substantia nigra
SNc: substantia nigra pars compacta
SNr: substantia nigra pars reticulata
