# Supplementary figures and images for: Amygdala activity for the modulation of goal-directed behavior in emotional contexts
Source: PLoS Biol. 2018 Jun 5;16(6):e2005339. doi: 10.1371/journal.pbio.2005339 (PMC5988268; doi:10.1371/journal.pbio.2005339)

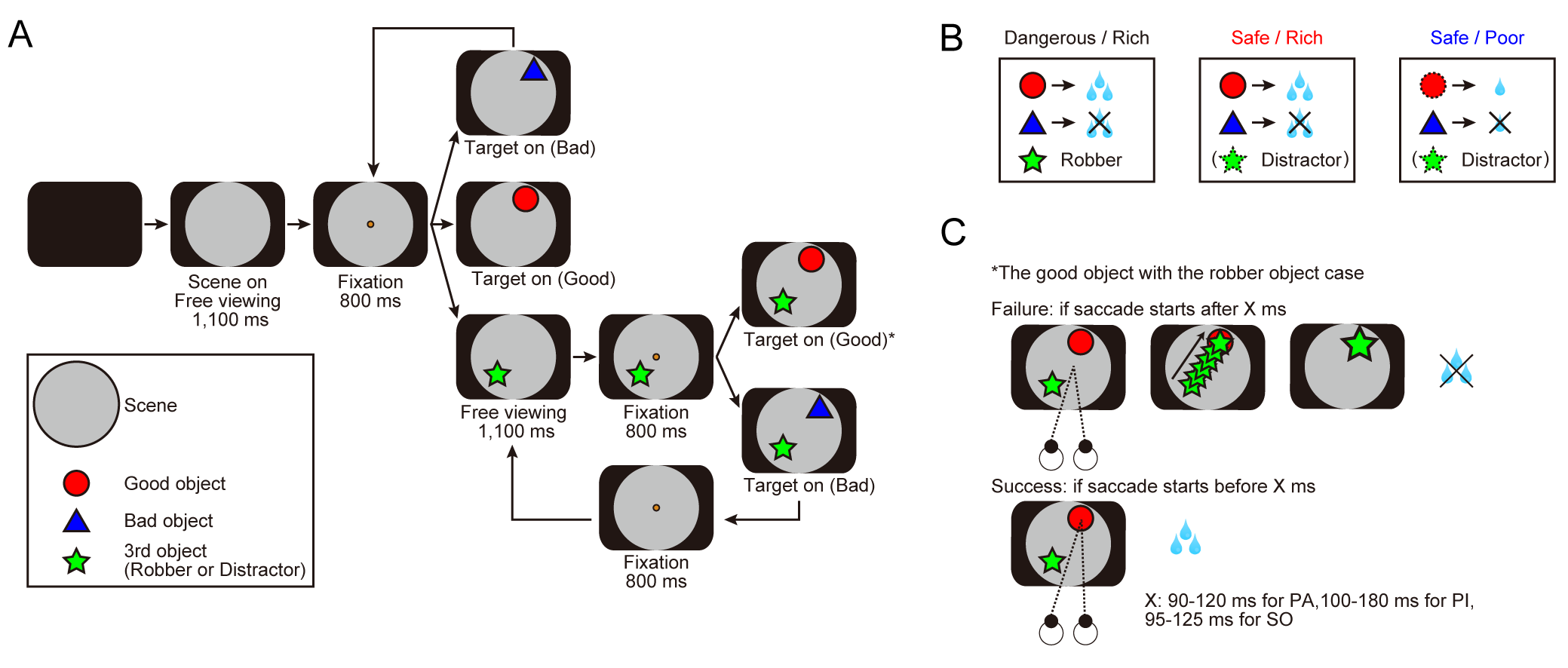

Supplement: S1 Fig — (A) The sequence of events on trials with additional (robber or distractor) object. An environment (scene) appears first, which contains two or three objects with meanings shown in (B). After gaze fixation at the center, one of the objects appears at a random position. A saccade to the good object followed by sustained gaze terminates the trial with delivery of reward. The amount of reward is either big or small depending on the scene, creating the rich versus poor dimension of context. Saccades to the bad object followed by sustained fixation terminate the trial with no reward. The subject thus learns to avoid the bad object by either withholding a saccade or leaving the bad object quickly, after which the fixation point reappears. Another object, either robber or distractor, remains for a while, irrespective of the animal’s behavior. The distractor simply remains on the screen, whereas the robber jumps to the good object (if present) and precludes reward delivery if it beats the monkey’s saccade (C, see S2 Movie). The presence or absence of the robber determines the dangerous versus safe dimension of context. Both dimensions of context were examined by varying the dimension of interest, while the orthogonal dimension was constant. See Fig 1 and Materials and methods. (TIF) [file pbio.2005339.s001.tif]

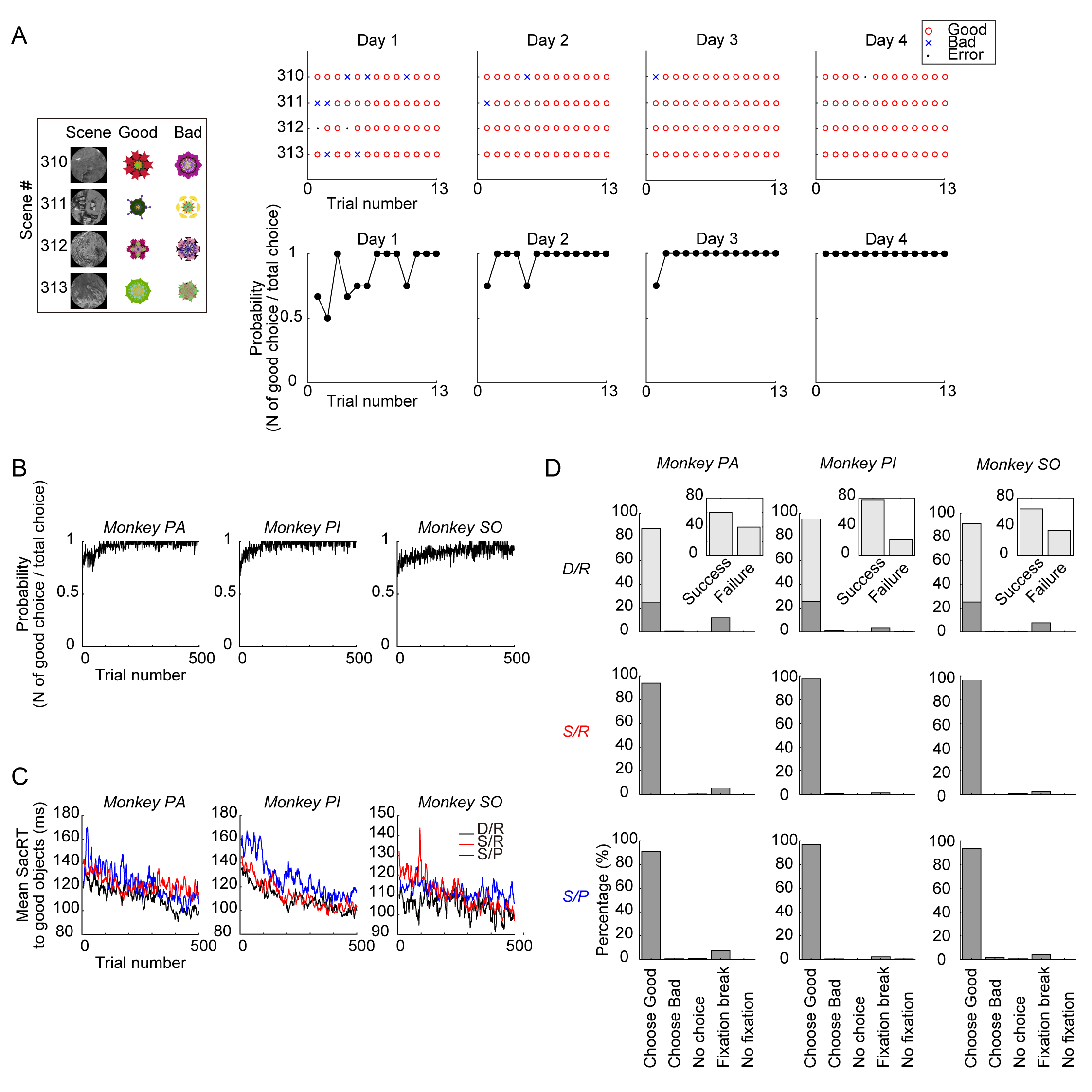

Supplement: S2 Fig — (A) Learning across 4-d sessions in monkey PI during the foraging task (4 scenes with 8 objects). Before this experiment the subject had learned the task rule completely, but all scenes and objects were completely new on Day 1. Each session consisted of 52 trials (13 trials for each scene). In the early stage of learning on Day 1, the subject’s choice was sometimes wrong (i.e., bad object) or invalid (i.e., fixation break) but became nearly perfect toward the end of the session. The speed of learning is shown as the change in the correct response rate. The good performance was well retained across days. These example scene images were derived from OpenAerialMap (https://openaerialmap.org). (B–D) Learning in three monkeys (PA, PI, SO). (B) Time course of object choice learning for many scenes (56 scenes for monkey PA and PI, 32 scenes for monkey SO). (C) Change in saccade reaction time to the good object across learning. For each subject, data are shown separately for three scene types: D/R, S/R, and S/P. Trial number was measured for individual scenes, not the subject’s task career. (D) Performance after learning (>200 trials for each scene) in the three groups of scenes. “Failure” indicates the D/R context trials in which the robber beat the monkey’s saccade. Dangerous trials featured the appearance of the robber; those that did are indicated in light gray. D/R, dangerous and rich; S/P, safe and poor; S/R, safe and rich. (TIF) [file pbio.2005339.s002.tif]

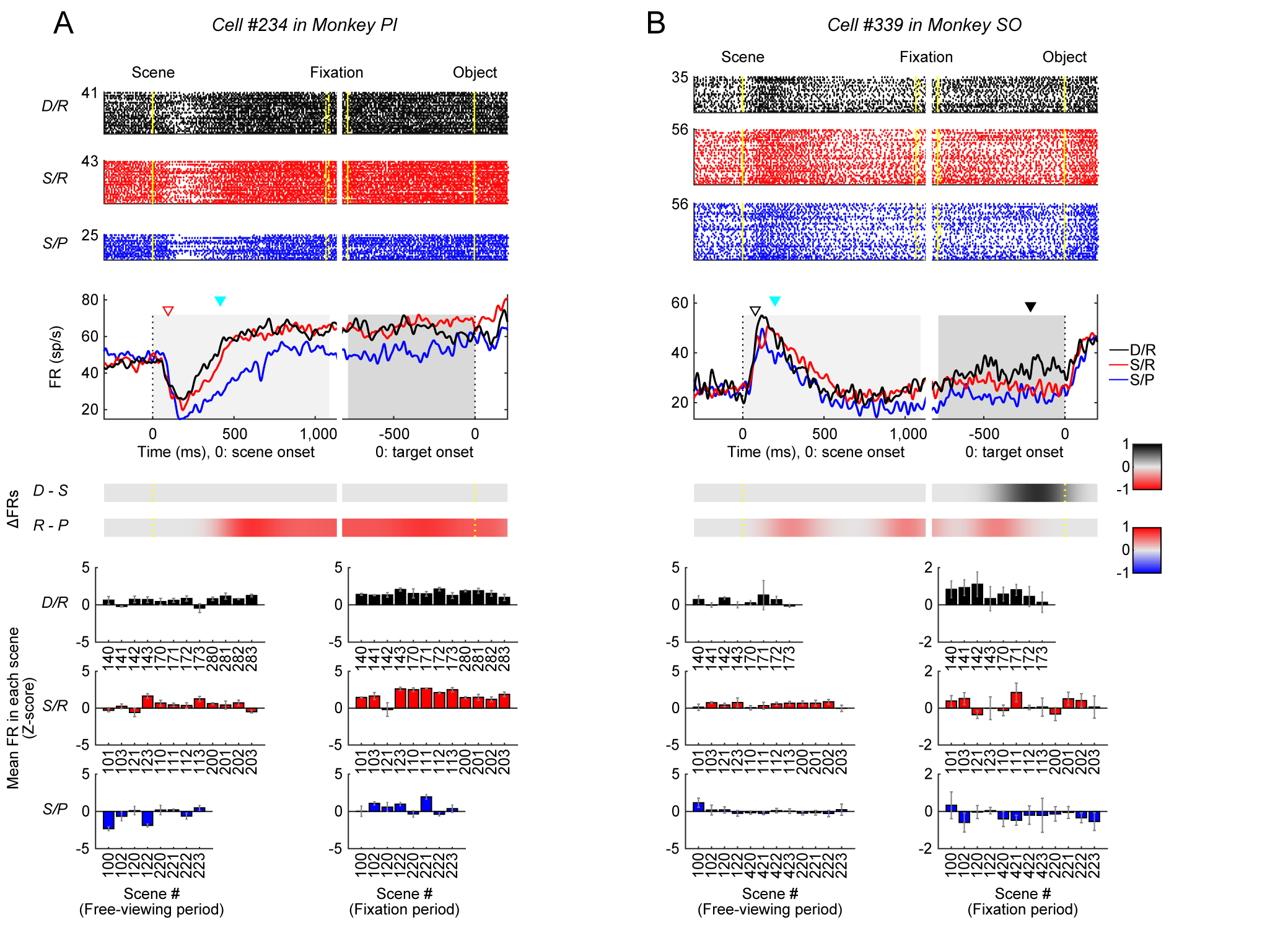

Supplement: S3 Fig — (A) Neuron in monkey PI (#234) that was selectively active in the rich context during the fixation period (S/R > S/P). (B) Neuron in monkey SO (#339) that was active in both the dangerous and rich contexts during the fixation period (D/R > S/R and S/R > S/P). The same format as in Fig 4. S/P, safe and poor; S/R, safe and rich. (TIF) [file pbio.2005339.s003.tif]

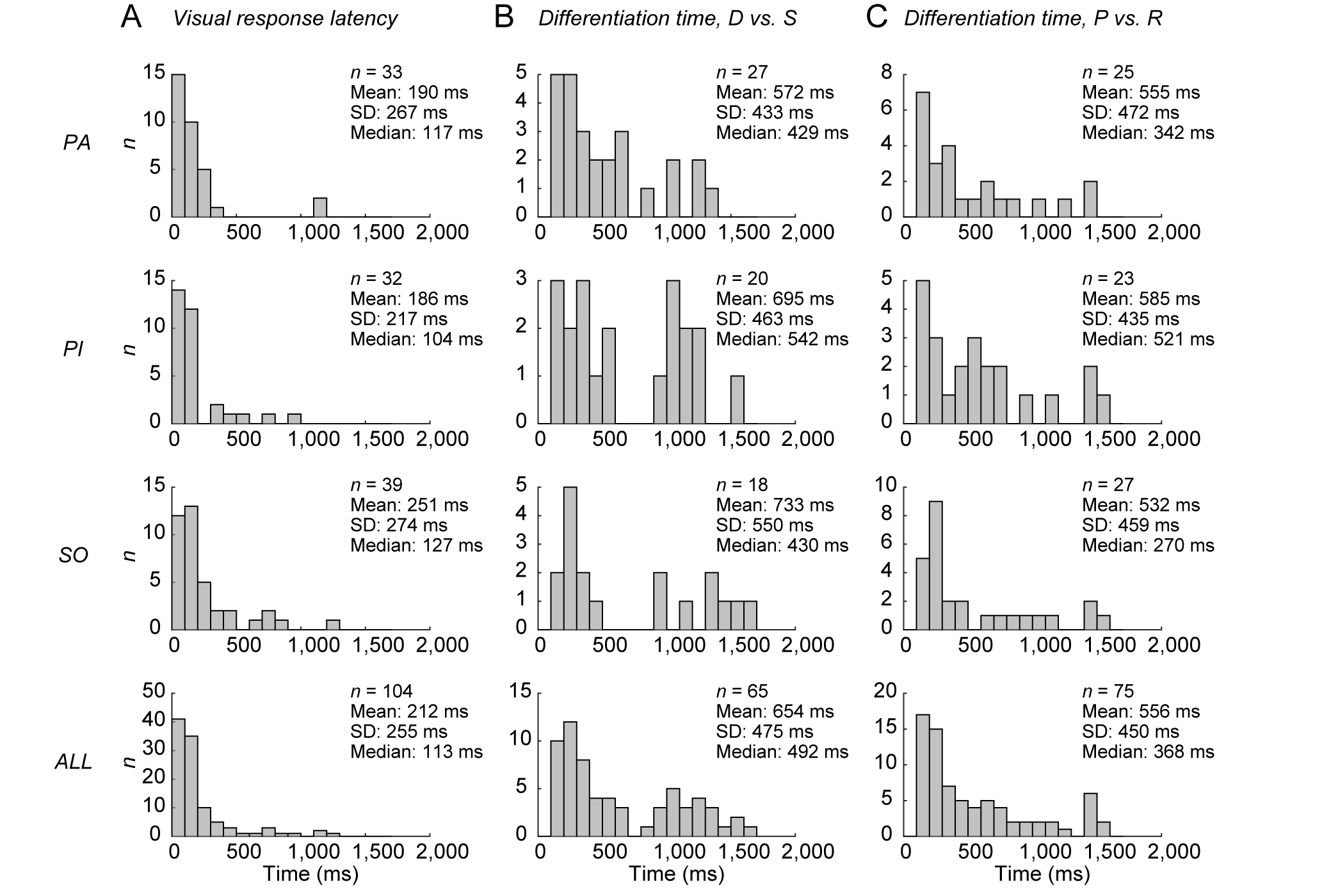

Supplement: S4 Fig — Latencies of neuronal responses to visual scenes. (A) General latency: time when the neuronal activity (PA, n = 33; PI, n = 32; SO, n = 39) changed significantly after the onset of any of the tested scenes. (B–C) Context-discrimination latency: time when the neuronal activity changed significantly between dangerous and safe scenes (B, PA, n = 27; PI, n = 20; SO, n = 18) and between rich and poor scenes (C, PA, n = 25; PI, n = 23; SO, n = 27). Data are based on excited-type neurons (see S4 Fig) in monkey PA, PI, SO, and all. (TIF) [file pbio.2005339.s004.tif]

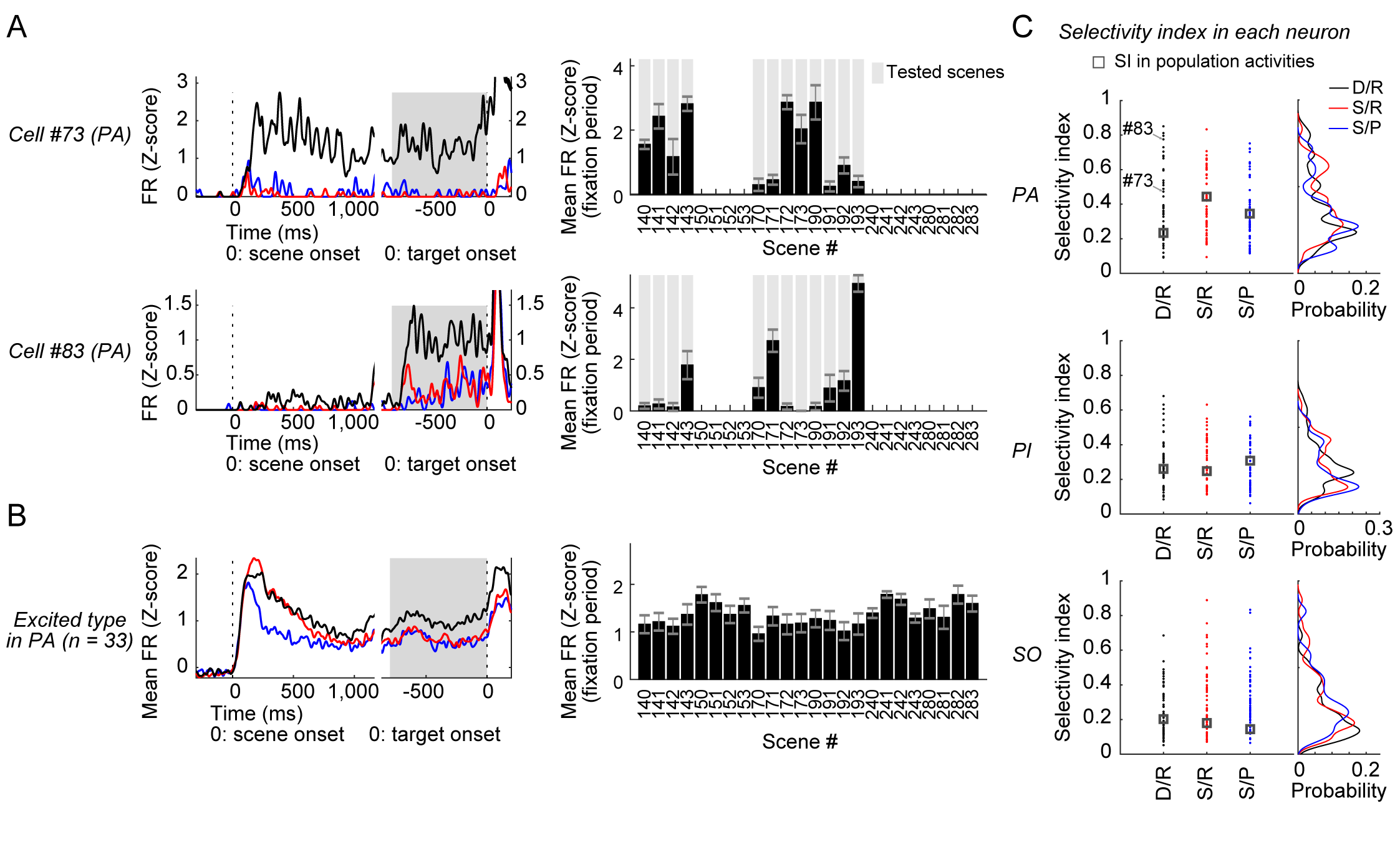

Supplement: S5 Fig — (A) Scene selectivity of two example neurons in monkey PA. Left: the neuron’s activities in the three groups of scenes: D/R, S/R, S/P. Right: the neuron’s responses to individual scenes in the dangerous (D/R) context (in the fixation period). Same format as in Fig 4B and 4D. Shaded gray area indicates the tested scenes for each neuron. Scene selectivity (SI): 0.473 (#73), 0.775 (#83). (B) Scene selectivity of the population neuronal activity (excited-type neurons, n = 33) in D/R context in monkey PA (SI: 0.234). Same format as in (A). (C) SIs in individual neuronal activity (small dots) and the population neuronal activity (gray squares) in three contexts in each monkey (PA, PI, SO). Mean SI: 0.386 (PA[D/R]), 0.404 (PA[S/R]), 0.345 (PA[S/P]), 0.299 (PI[D/R]), 0.298 (PI[S/R]), 0.264 (PI[S/P]), 0.245 (SO[D/R]), 0.305 (SO[S/R]), 0.311 (SO[S/P]). Population SI: 0.234 (PA[D/R]), 0.444 (PA[S/R]), 0.346 (PA[S/P]), 0.261 (PI[D/R]), 0.248 (PI[S/R]), 0.308 (PI[S/P]), 0.203 (SO[D/R]), 0.180 (SO[S/R]), 0.145 (SO[S/P]). On the right, the probabilistic distribution of SIs is shown by kernel density estimation (width = 0.03) for each context and in each monkey. D/R, dangerous and rich; S/P, safe and poor; S/R, safe and rich; SI, selectivity index. (TIF) [file pbio.2005339.s005.tif]

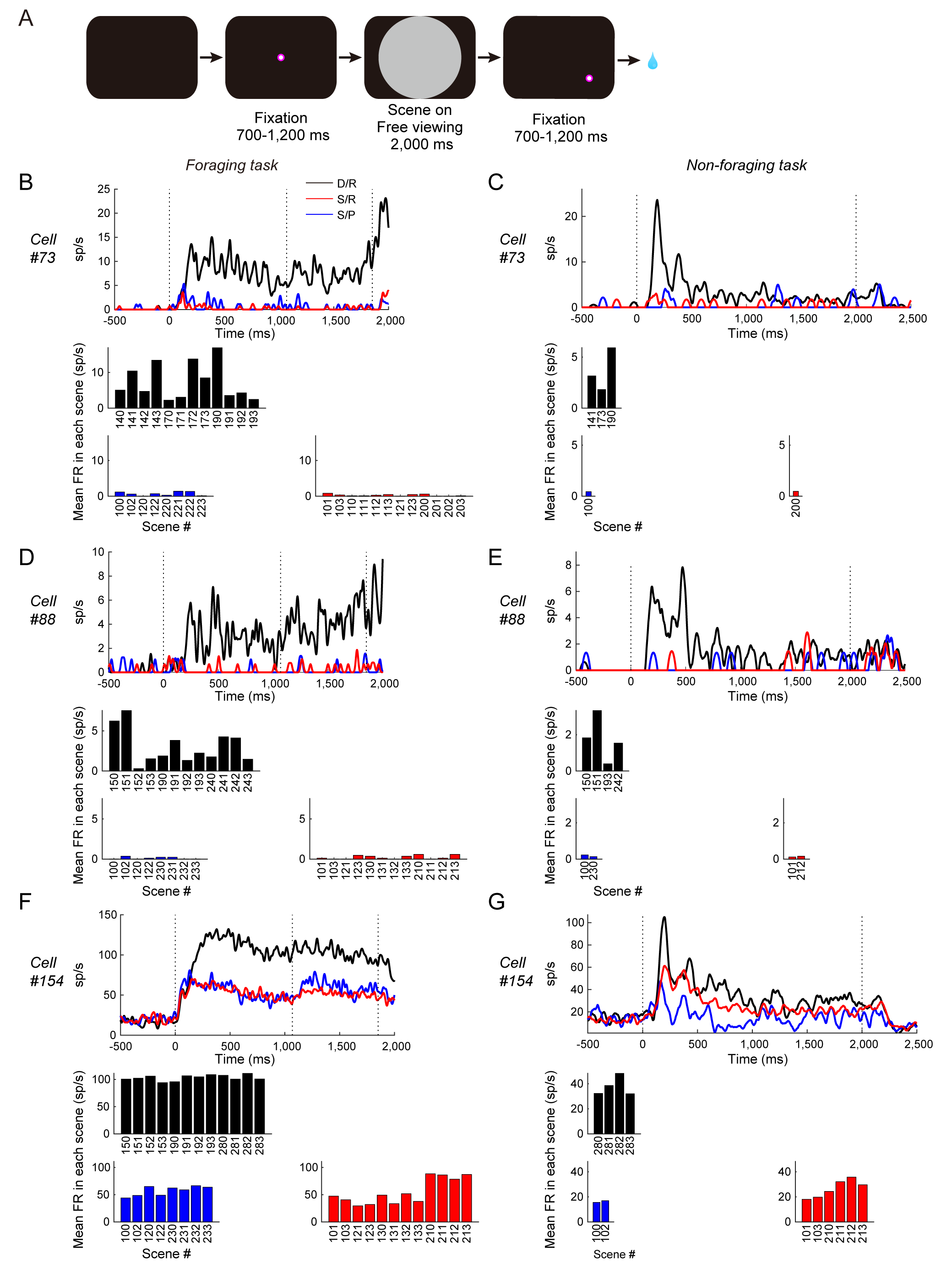

Supplement: S6 Fig — (A) Nonforaging task. Reward was delivered if the subject kept gaze on the fixation point that moved from the center to a random peripheral position. Before the position change, a well-learned scene was presented for 2,000 ms, during which free viewing was allowed. Across trials, different scenes appeared randomly, but no object (good, bad, robber, distractor) appeared inside the scene. (B–G) Activity of three neurons in monkey PA during the foraging task (B, D, F) and nonforaging task (C, E, G). These tasks were presented as separate blocks of trials. Their responses to individual scenes are also shown. The FV and FX period in the foraging task was combined. FV, free-viewing; FX, fixation. (TIF) [file pbio.2005339.s006.tif]

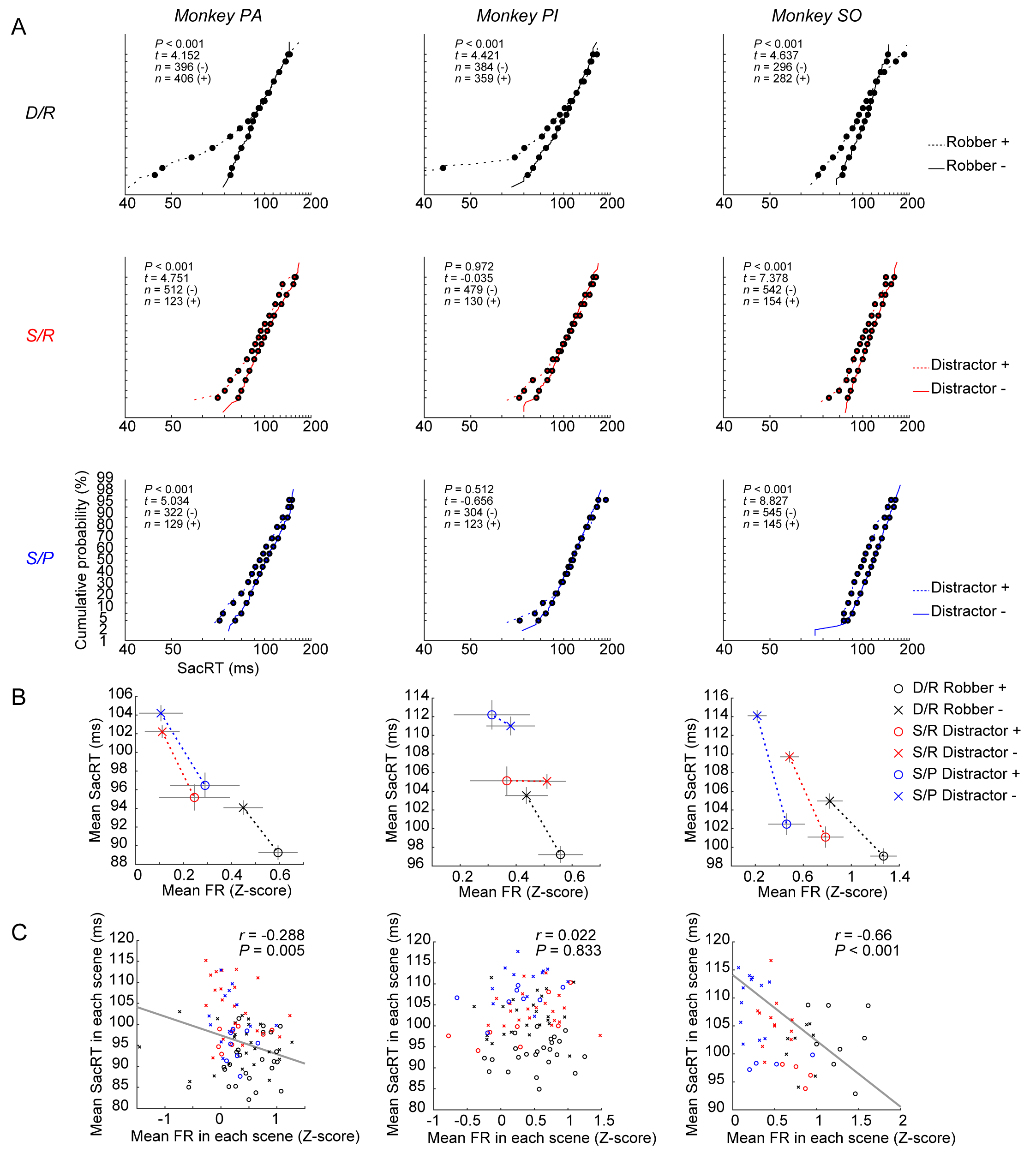

Supplement: S7 Fig — (A) Distributions of the SacRT to good objects in three monkeys (PA, PI, SO) in three groups of scenes: S/P, D/R, S/R. In each panel, data from two groups of object context are superimposed: Robber(+) and Robber(−) in D/R, and Distractor(+) and Distractor(−) in S/R and S/P. SacRT distribution is shown using reciprobit plot. (B) Relation between the neuronal activity (abscissa) and SacRT (ordinate) in two dimensions of context: environment contexts (D/R, S/R, S/P) and object context (Robber/Distractor[+], Robber/Distractor[−]). The object contexts shown here correspond to data shown in (A). (C) Relation between the neuronal activity (abscissa) and SacRT (ordinate) for individual scenes. Some scenes provide two data points: Robber/Distractor(+) and Robber/Distractor(−). Findings are presented in further detail below. In the dangerous scene (D/R), SacRT was shorter when a robber object was present (Robber+) than when absent (Robber−) (B). The distribution of SacRT became curved, indicating that the saccade preparation process became non-Gaussian by including extremely short SacRTs (A, D/R). Notably, these effects occurred in the all monkeys. Taken together with data shown in B, these results show that all monkeys were sensitive to danger, in both scene and object (i.e., robber present or absent) contexts. Monkey PI was primarily sensitive to the object context (faster when a robber object was present), in tandem with amygdala neurons tending to be more active on Robber(+) trials than on Robber(−) (B), although the statistical significance was shown only in monkey SO. Results of two-way ANOVA tests with environments and object+/− with Tukey–Kramer post hoc tests were as follows. Monkey PA: F[2, 1882] = 0.025, P = 0.975, post hoc, P = 0.811. Monkey PI: F[2, 1773] = 1.149, P = 0.317, post hoc, P = 0.882. Monkey SO: F[2, 1958] = 0.425, P = 0.654, post hoc, P = 0.0422. In the safe scenes (S/R, S/P), SacRT was shorter when a distractor object was present than when absent in [file pbio.2005339.s007.tif]

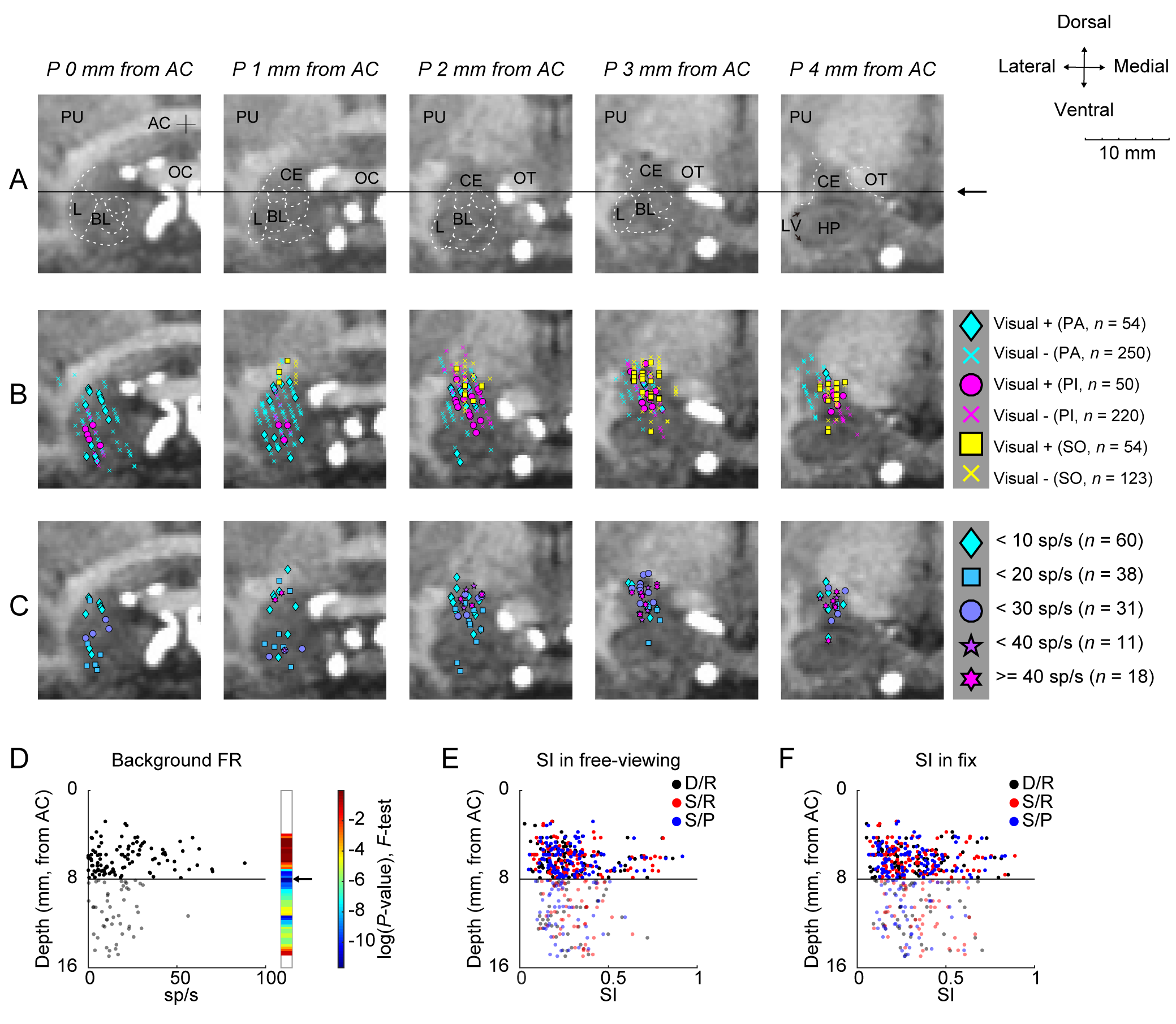

Supplement: S8 Fig — (A) Amygdala and surrounding brain areas. Same format as in Fig 8A. A horizontal black line indicates the dorsoventral border of neurons based on their background firing rate (data shown in D). (B) Neurons with visual responses (Visual +) and with no visual responses (Visual −), shown separately for three monkeys. (C) Visual scene-sensitive neurons (excited type) are classified based on their background FRs. (D) Background FRs of individual neurons (abscissa) plotted against their recorded depths from the AC (ordinate). The horizontal line (i.e., 8 mm below AC) indicates the dorsoventral border, by which the variance of Background FR was maximally higher in the dorsal area than the ventral area. This was determined by the lowest P value (two-sample F-test) while moving the border line (0.2 mm step) (as shown in colored bars on the right) (F[108, 48] = 3.363, P < 0.001, dorsal [355.856], ventral [105.812]). Note that the border line roughly corresponds to the border between CE and BL/L. (E-F) SIs of individual neurons (abscissa) plotted against their recorded depths (ordinate) during the free-viewing period (E) and the fixation period (F). With the border based on Background FR (D), the variance of SIs was significantly higher in the dorsal area than the ventral areas (SI in free-viewing: F[324,146] = 1.958, P < 0.001, dorsal [0.030], ventral [0.015]; SI in fixation: F[326,146] = 1.476, P = 0.008, dorsal [0.033], ventral [0.023]). AC, anterior commissure; BL/L, basolateral complex and lateral nucleus of the amygdala; CE, central nucleus of the amygdala; FR, firing rate; SI, Selectivity index. (TIF) [file pbio.2005339.s008.tif]
